# DTBind: A Mechanism-Driven Deep Learning Framework for Accurate Prediction of Drug–Target Molecular Recognition

**DOI:** 10.34133/research.1022

**Published:** 2025-12-02

**Authors:** Qiuyu Li, Zeyu Xu, Yanhao Zhu, Wanyun Zhou, Mingming Guan, Shiqing Zhao, Mengyuan Liu, Bin Liu, Juntao Liu

**Affiliations:** ^1^ School of Mathematics and Statistics, Shandong University, Weihai 264209, China.; ^2^ The Hong Kong University of Science and Technology (Guangzhou), Guangzhou 511453, China.; ^3^ Department of Neurosurgery, Shandong Provincial Hospital Affiliated to Shandong First Medical University, Jinan 250021, China.

## Abstract

Accurate prediction of drug–target molecular recognition is essential for early-stage drug discovery, spanning binding occurrence, binding site localization, and binding affinity estimation. However, current approaches frequently treat these tasks independently, thereby overlooking the shared mechanistic principles that underlie them. We present DTBind, a unified and mechanism-driven framework that hierarchically adapts to sequence, structure, and complex-level inputs for predicting binding occurrence, site, and affinity, grounded in the shared mechanistic determinants of molecular recognition. Comprehensive benchmarking demonstrates that DTBind achieves superior performance over state-of-the-art methods in terms of accuracy and generalizability. Analysis of hierarchical protein encodings demonstrates that determinant-based protein representations gradually drive the accurate prediction of molecular recognition. Validation based on molecular dynamics simulations shows that DTBind reliably predicts drug–target binding regions for 3 famous proteins that lack experimental structures.

## Introduction

Molecular recognition denotes the process in which 2 or more molecules achieve specific binding through noncovalent interactions such as hydrogen bonding, van der Waals forces, metal coordination, hydrophobic interactions, and π–π stacking [1–4]. In drug discovery, this process embodies 3 interrelated levels of understanding: whether binding occurs, where it takes place, and how strong the interaction is. These questions represent different levels of analyzing a single, coherent recognition process rather than isolated computational tasks. Predicting binding occurrence enables efficient virtual screening [5–7]; identifying binding sites offers structural insights for lead optimization [8–11]; and estimating binding affinity quantifies compound potency [12–14]. Critically, they are manifestations of molecular recognition governed by shape complementarity, functional group matching, physicochemical compatibility, and surface accessibility [9,15–17]. A unified understanding of these aspects offers a complete view of molecular recognition that supports rational drug design and accelerates early-stage discovery.

Traditional analytical techniques such as x-ray crystallography, nuclear magnetic resonance (NMR), and mass spectrometry provide high-resolution insights into molecular recognition but are time-consuming, costly, and difficult to scale [18–20]. Molecular dynamics (MD) simulations can capture atomistic dynamics and reveal conformational flexibility and induced fit during binding, but they rely on high-quality 3-dimensional (3D) structures and are computationally expensive, limiting their applicability for large-scale screening [21,22]. Consequently, machine learning and deep learning approaches have emerged as scalable and efficient alternatives [15,23–41].

However, current computational approaches often fragment the integrated process of molecular recognition into isolated tasks, overlooking the nature that binding occurrence, binding site localization, and affinity estimation form a hierarchical continuum—progressing from coarse-grained detection to fine-grained localization, and finally to quantitative refinement. This conceptual fragmentation has led to rigid, single-modality models constrained by fixed input–output mappings, failing to flexibly adapt to the distinct data demands across hierarchical predictions. While binding occurrence can often be inferred from sequence (e.g., DrugBAN [28] and MolTrans [42]), finer-resolution predictions such as site or affinity prediction generally require structural data (e.g., DeepSurf [32] and KDBNet [43]). This division has led to model architectures that are siloed by modality: They either fail to exploit available structural information for deeper insights or are inapplicable when such information is missing, consequently providing only a fragmented view of the recognition process. Although these predictive levels differ in data requirements and predictive granularity, they are fundamentally governed by the common recognition mechanism. Therefore, hierarchical modeling grounded in shared mechanistic determinants and adaptable to diverse input conditions essential for achieving a unified understanding of molecular recognition.

Beyond the aforementioned issues of architectural fragmentation, a major limitation at the model design level is the failure to holistically incorporate the key determinants underlying molecular recognition. While encoders of existing methods learn representations from sequences, graphs, or structures, few models systematically incorporate determinants such as geometric fit, cooperative interactions, or surface morphology. MolTrans [42] enhances sequence modeling through substructure-level Transformers yet still depends solely on correlation patterns without geometric awareness. DeepDTA [25] employs convolutional neural networks (CNNs) on sequence encodings to predict binding strength but lacks spatial complementarity and surface accessibility information essential for physical binding feasibility. KDBNet [43] integrates pocket-level structures with graph neural networks (GNNs) to estimate affinity, yet omits surface accessibility that critically determines ligand accommodation. Overall, these methods isolate key determinants and fail to capture their coordinated effect, which limits their generalization across the multiple levels of molecular recognition prediction.

To address these limitations, we propose DTBind, a unified, mechanism-driven framework for drug–target molecular recognition that hierarchically predicts binding occurrence, binding sites, and binding affinity. DTBind explicitly encodes the key determinants of recognition into both protein and drug representations. Proteins are modeled as residue-level graphs, with each residue represented by local embeddings capturing geometry, physicochemical and functional properties, and surface features encoding pocket morphology and accessibility. Drugs are represented as atom-level graphs that encode atomic and bond-level physicochemical attributes. The shared, determinant-aware encoder integrates these representations, which are then interpreted by 3 information-adaptive decoders: The binding occurrence decoder focuses on global intermolecular features to determine whether binding occurs, the binding site decoder emphasizes residue-level distinctions to identify interacting regions, and the binding affinity decoder models atom–residue interactions to estimate binding strength. By coupling the consistent mechanistic principles across sequence, structure, and complex data, DTBind provides a coherent, unified framework for generalizable prediction of molecular recognition.

Extensive experiments demonstrate that DTBind consistently surpasses baseline methods in both predictive accuracy and robustness. Hierarchical protein encoding analysis reveals that determinant-based protein features make indispensable contributions by progressively enhancing the accurate prediction of molecular recognition. Evaluations across diverse complexes demonstrate that DTBind accurately captures all 3 predictive levels, encompassing binding occurrence, binding site localization, and binding affinity estimation. MD-based validation further shows that DTBind reliably provides binding regions for 3 well-known drug–target pairs lacking experimental structures. Collectively, DTBind establishes a unified and determinant-aware framework that not only assists early-stage drug screening but also advances a systematic and mechanism-grounded understanding of drug–target molecular recognition.

## Results

### Overview of the DTBind framework

**Fig. 1.**
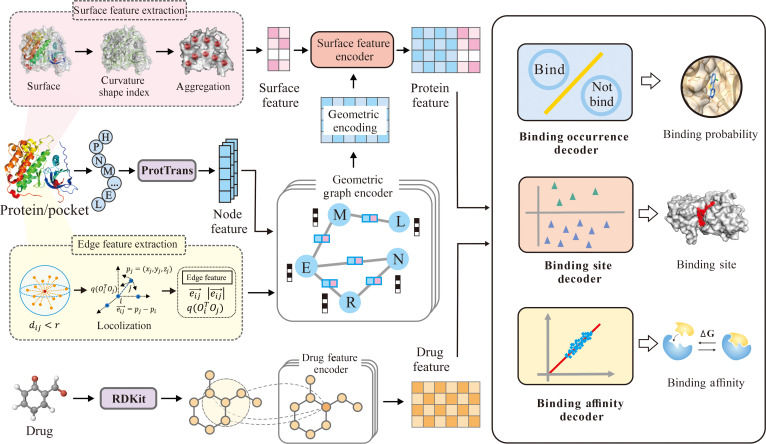
The DTBind framework. DTBind provides a unified framework for drug–target molecular recognition. The architecture includes the following: (1) Drugs are modeled as atom-level graphs, and targets as both residue-level graphs and surface representations; (2) Drug feature extraction as molecular graphs with physicochemical properties of atoms and chemical bonds; (3) Protein and drug encoders that generate residue- and atom-level embeddings; and (4) Information-adaptive decoders for binding occurrence prediction, site identification, and affinity estimation.

**Fig. 2.**
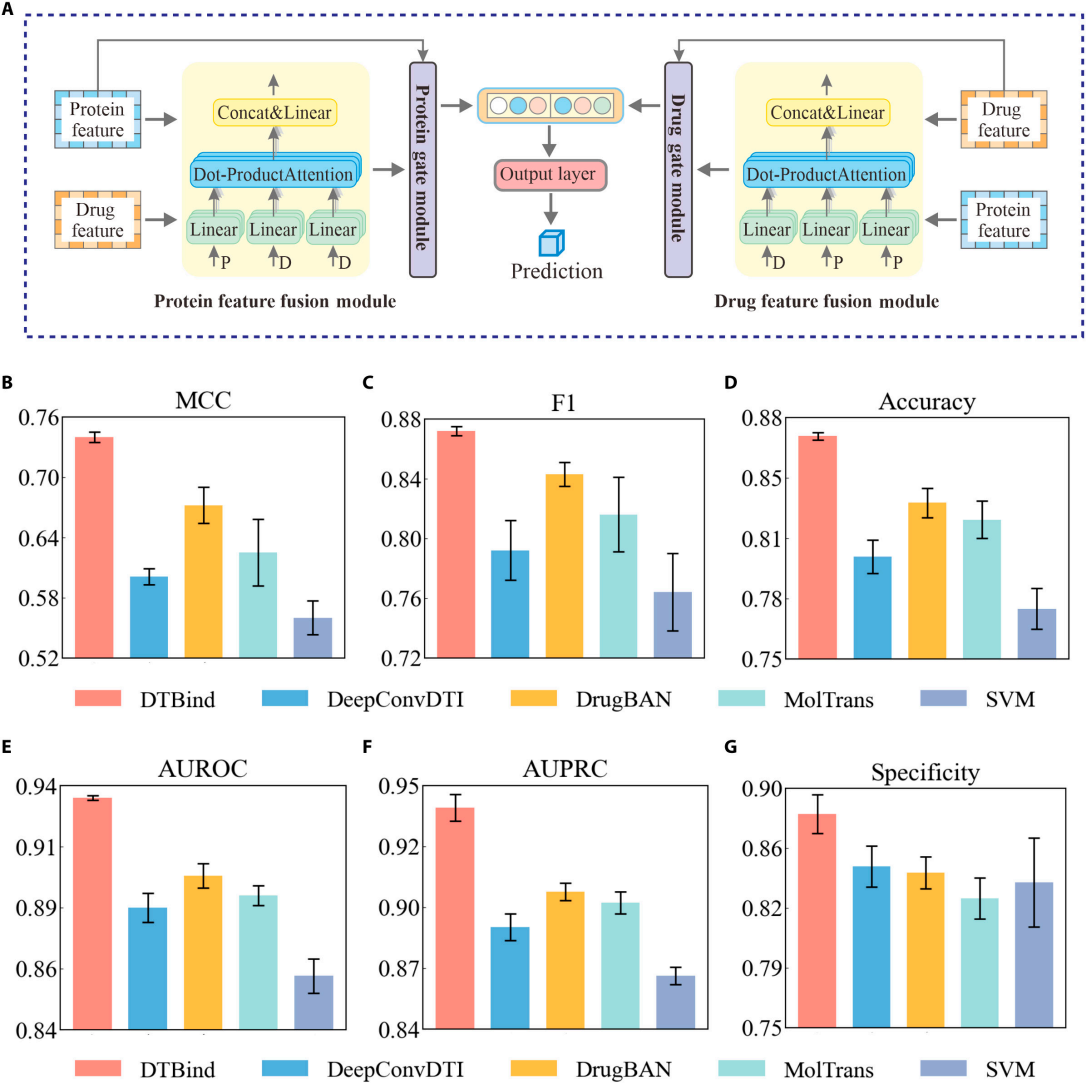
Overview of the binding occurrence decoder and the performance comparison of binding occurrence prediction. (A) Architecture of the binding occurrence decoder fusing drug and protein embeddings via a multi-head cross-attention with gating, followed by pooling and output layer output. (B to G) Comparisons with baselines (DeepConvDTI [45], DrugBAN [28], MolTrans [42], and SVM [46]) on MCC (B), F1-score (C), Accuracy (D), AUROC (E), AUPRC (F), and Specificity (G). The height of each bar represents the mean value over 5 independent runs with different random seeds, and the black vertical lines denote the standard deviation across 5 runs.

**Fig. 3.**
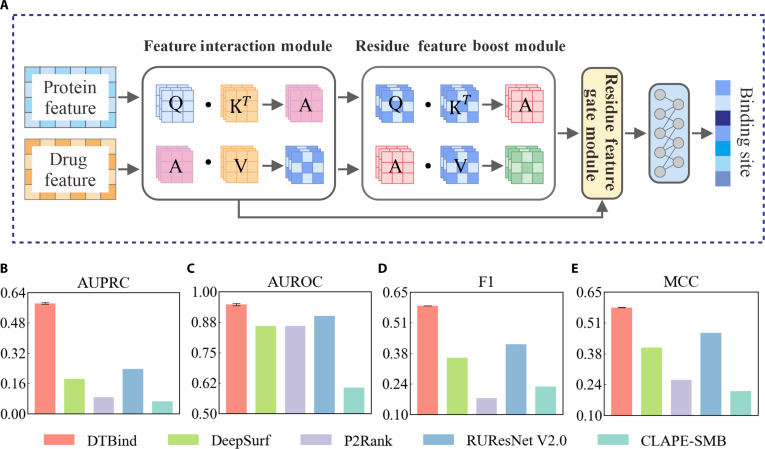
Overview of the binding site decoder and performance comparison of binding site prediction. (A) Architecture of the binding site decoder including residue-level protein features with drug representations via cross-modal attention, self-attention mechanism, and classify layer. (B to E) Comparisons with baselines including DeepSurf [32], P2Rank [48], PUResNet V2.0 [27], and CLAPE-SMB [49] in terms of AUPRC (B), AUROC (C), F1-score (D), and MCC (E). The height of each bar represents the mean value over 5 independent runs with different random seeds, and the black vertical lines denote the standard deviation across 5 runs.

**Fig. 4.**
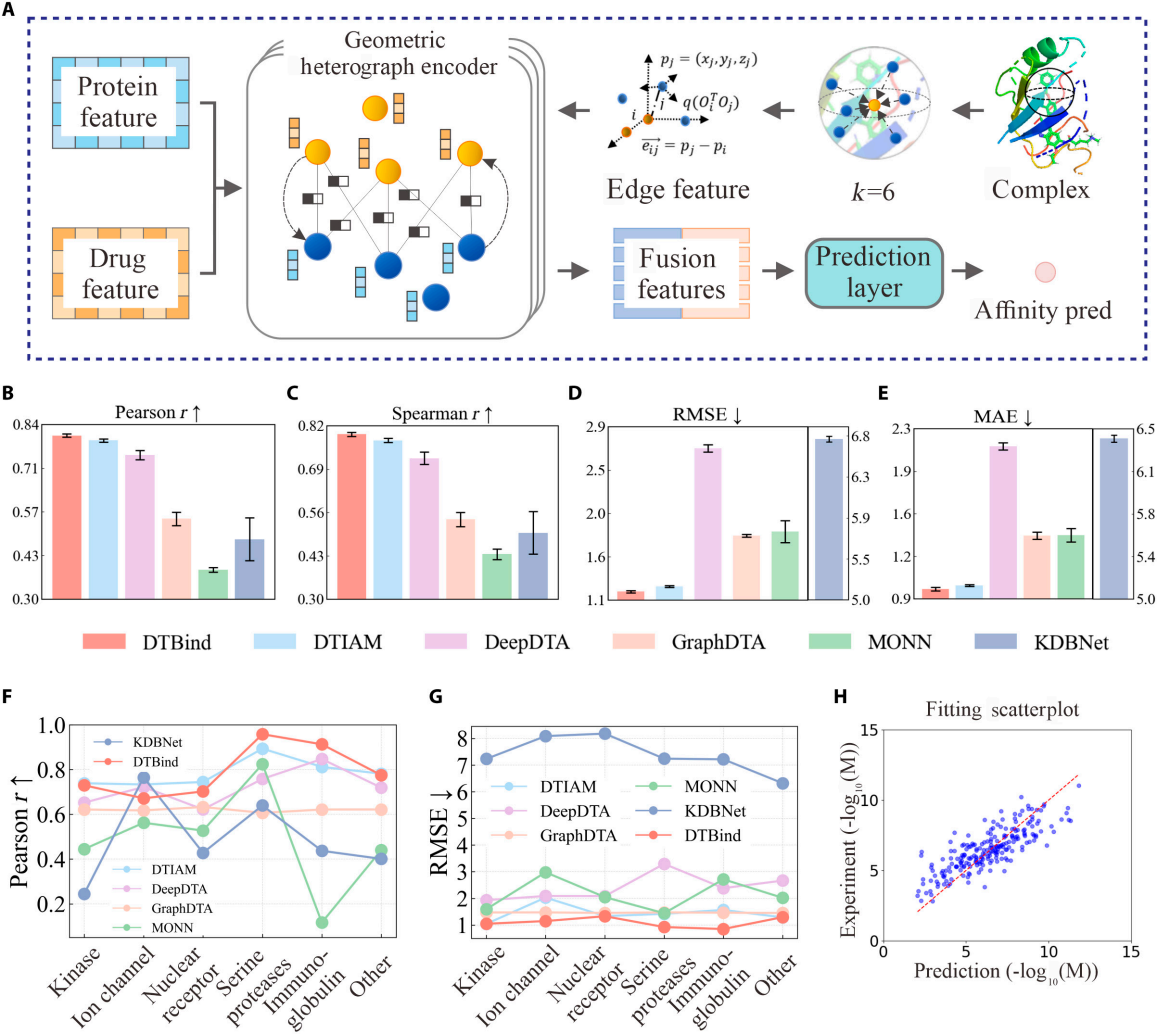
Overview of the binding affinity decoder and performance comparison of binding affinity prediction. (A) Detailed architecture of the binding affinity decoder. Protein and drug features are embedded into a heterogeneous graph; heterogeneous graph convolution captures intermolecular dependencies, and fused features are mapped by prediction layers to a continuous affinity score. (B to E) Performance comparisons with DTIAM [50], DeepDTA [25], GraphDTA [29], MONN [51], and KDBNet [43] on Pearson (B), Spearman (C), RMSE (D), and MAE (E). The height of each bar represents the mean value over 5 independent runs with different random seeds, and the black vertical lines denote the standard deviation across 5 runs. Full numerical results are provided in Table S4. (F and G) Generalization across 6 protein families (kinase, nuclear receptor, ion channel, immunoglobulin, serine proteases, other) demonstrates DTBind’s best or near-best Pearson (F) and RMSE (G) in each family. (H) Scatterplot of predicted versus experimental affinities, illustrating close agreement.

The architecture of DTBind (Fig. 1) provides a unified, determinant-aware framework for drug–target molecular recognition, in which a shared, determinant-aware encoder produces molecular representations of proteins and drugs that are then interpreted by information-adaptive decoders to perform sequence-driven binding occurrence, structure-guided binding site localization, and complex-informed binding affinity estimation. Drugs are represented as atom-level molecular graphs encoding the physicochemical properties of atoms and chemical bonds, whereas protein targets are modeled as residue-level graphs with layered embeddings capturing geometry, functional and physicochemical properties, and surface features encoding pocket morphology and accessibility. These molecular graphs are encoded to update atom- and residue-level embeddings, which are subsequently fused and passed to information-adaptive decoders aligned with the 3 recognition dimensions: binding occurrence (Fig. 2A), binding site localization (Fig. 3A), and binding affinity estimation (Fig. 4A).

The binding occurrence decoder (Fig. 2A) employs a dual-channel cross-attention mechanism to capture global drug–target interactions, with separate pathways for drug-to-protein and protein-to-drug attentions. Gating mechanisms are applied to each channel to selectively refine and emphasize the most informative features in the fused embeddings for classification. The binding site decoder (Fig. 3A) applies a multi-head cross-attention (MCA) to capture fine-grained residue–drug interactions and a residue self-attention to enhance residue-specific context. Then, gated layers adaptively retain the learned information for producing residue-level binding probabilities. The binding affinity decoder (Fig. 4A) represents each drug–target complex as a heterogeneous molecular graph with nodes representing protein residues and drug atoms, and edges encoding residue–atom interaction information. Attention-weighted residue–atom pairwise interactions are fused with global protein and drug embeddings, capturing both local and global molecular interplay to yield accurate binding affinity estimation.

### DTBind accurately predicts drug–target binding based on target sequences

Determining whether a drug can bind a candidate protein target represents the entry point of drug–target recognition. Accurate prediction of this binary outcome is essential for prioritizing viable candidates and focusing downstream analysis on meaningful interactions. Accordingly, binding occurrence prediction is formulated as a binary classification problem and a dedicated prediction module is implemented accordingly (Methods; Fig. 1, binding occurrence prediction module, and Fig. 2A). We evaluated DTBind on the BioSnap [44] benchmark dataset, where positive and negative drug–target pairs are nearly balanced at a 1:1 ratio. The dataset was divided into training, validation, and test sets following the procedure detailed in Methods. We evaluated DTBind against representative baseline methods—DeepConvDTI [45], DrugBAN [28], MolTrans [42], and support vector machine (SVM) [46]. To comprehensively assess the predictive performance under this balanced setting, we report Matthews correlation coefficient (MCC), F1-score, Accuracy, area under the receiver operating characteristic curve (AUROC), area under the precision–recall curve (AUPRC), and Specificity as representative metrics (Fig. 2B to G), and detailed metrics are provided in Tables S1 and S2. These 6 metrics together provide a comprehensive evaluation, capturing not only ranking ability (AUROC and AUPRC) and balanced discrimination between positive and negative classes (F1-score and MCC) but also overall correctness (Accuracy) and negative-class resolution (Specificity).

Across all baselines, DTBind consistently achieved superior and more stable performance, as evidenced by both higher mean scores and smaller variances over 5 independent runs with different random seeds. Compared to DeepConvDTI [45], MolTrans [42], and a conventional SVM [46] with handcrafted features, DTBind showed substantial improvements across all 6 metrics (Fig. 2B to G). Among these baselines, DrugBAN [28] emerged as the strongest competitor, benefiting from its bilinear attention for cross-modal alignment, yet DTBind still surpassed it. Specifically, DTBind achieved an AUROC of 0.935 and AUPRC of 0.940, exceeding DrugBAN [28] by +3.54% in AUROC and +4.21% in AUPRC. On balanced discrimination, DTBind reached an F1-score of 0.872 and MCC of 0.740, corresponding to +3.44% in F1 and +10.12% in MCC over DrugBAN [28]. These consistent improvements further extended to Accuracy (+4.32% over DrugBAN) and Specificity (+4.37% over DrugBAN), highlighting not only DTBind’s ability to discriminate true binders from nonbinders but also its robustness in predicting both positive and negative pairs. Together, these results demonstrate that DTBind provides a more reliable prediction of drug–target interactions than state-of-the-art baselines.

### DTBind accurately identifies binding sites based on protein structures

Beyond the occurrence of binding, identifying binding regions provides the structural context necessary for mechanistic interpretation and rational optimization. Accurate binding site prediction pinpoints residue-level determinants that guide both functional understanding and design strategies. The residue-level binding site identification is a highly imbalanced binary classification problem, where only a small fraction of residues serve as true binding sites. Accordingly, we designed a dedicated binding site decoder (Methods; Fig. 1, binding site prediction module, and Fig. 3A). We evaluated DTBind on the PDBbind dataset [47], with dataset preparation and partitioning detailed in Methods. For comparison, we benchmarked DTBind against 4 state-of-the-art approaches—DeepSurf [32], P2Rank [48], PUResNet V2.0 [27], and CLAPE-SMB [49]. Performance was assessed using F1-score, MCC, AUROC, and AUPRC as primary metrics (Fig. 3B to E), and detailed metrics are reported in Table S3. The 4 metrics collectively reflect the model’s comprehensive performance in an imbalanced problem.

DTBind was evaluated over 5 independent runs with different random seeds, yielding highly stable results on the PDBbind [47] test set (F1 = 0.589 ± 0.001, MCC = 0.581 ± 0.001, AUROC = 0.948 ± 0.005, AUPRC = 0.584 ± 0.005). By contrast, existing binding site predictors such as DeepSurf [32], P2Rank [48], PUResNet V2.0 [27], and CLAPE-SMB [49] provide only single-run results obtained directly from their pretrained models or online servers released by their authors. The absence of retraining stems from the lack of publicly available training code and/or data preprocessing explanation in these baseline methods. DTBind’s performance, evaluated over 5 independent runs, exhibited very low variance, with improvements over the baselines far exceeding this variance. Under these conservative conditions, DTBind consistently outperforms all baseline methods across all 4 evaluation metrics (Fig. 3B to E). For example, DTBind improved F1-score by +41.3% over PUResNet V2.0 [27] (the second best predictor), and by as much as +65.8% over DeepSurf and +239.8% over P2Rank [48]. MCC exhibited a similar trend, with +24.3% over PUResNet V2.0 [27] and up to +178.9% over CLAPE-SMB [49]. In ranking-based evaluation, DTBind achieved an AUROC of 0.948, exceeding PUResNet V2.0 [27] by +5.3% and CLAPE-SMB [49] by +56.6%, while its AUPRC reached 0.584—an improvement of +145.8% over PUResNet V2.0 [27] and more than +500% over P2Rank [48] and CLAPE-SMB [49]. These gains highlight that properly modeling drug–target interactions in a mechanism-driven manner allows DTBind to more effectively identify binding and nonbinding residues. These results establish DTBind as a more reliable and stable framework for residue-level binding site identification.

### DTBind accurately estimates drug–target binding affinity based on complex structures

Drug–target recognition must be quantified by binding strength, as affinity determines potency, dosing feasibility, and therapeutic viability. Reliable affinity prediction transforms qualitative recognition into a quantitative pharmacological measure. To this end, we designed a dedicated binding affinity decoder (Methods; Fig. 1, binding affinity decoder, and Fig. 4A). The drug–target affinity prediction is formulated as a regression task on the PDBbind dataset [47] (dataset preparation and partitioning detailed in Methods, “Data preparation” section). We evaluated DTBind against 5 representative baseline methods—DTIAM [50], DeepDTA [25], GraphDTA [29], KDBNet [43], and MONN [51]—on the CASF-2016 benchmark [52] (285 high-quality protein–ligand complexes extracted from the PDBbind v2016 refined set), with all models trained and tested over 5 independent runs to ensure robustness and reproducibility. Performance was assessed using root mean square error (RMSE) and mean absolute error (MAE) for absolute predictive accuracy and Pearson and Spearman correlations for linear and rank-order consistency.

After comparison, DTBind consistently outperformed all baselines across all metrics (Fig. 4B to E; full numbers in Table S4): The mean RMSE was 1.2393, representing a reduction of ~3.7% relative to the closest competitor DTIAM [50]; the mean MAE was 0.9810, a decrease of ~2.8%. Correlation metrics were also markedly improved, with a mean Pearson coefficient of 0.8042 (+1.7%) and a mean Spearman coefficient of 0.7947 (+2.7%) compared to DTIAM [50]. Compared with GraphDTA [29], DeepDTA [25], MONN [51], and KDBNet [43], DTBind achieved substantial error reductions (30.6% to 81.8% in RMSE and 30.9% to 84.7% in MAE) while markedly enhancing correlation (Pearson by 8.0% to 106.1% and Spearman by 10.0% to 82.8%), establishing consistent advantage across all benchmarks. A scatterplot of predicted versus experimental affinities for the entire test set (Fig. 4H) shows that DTBind predictions are distributed closely around the *y* = *x* line, reflecting strong consistency with experimental measurements. These results indicate that DTBind achieves substantially lower prediction errors while yielding stronger linear and rank-order consistency, thereby offering more accurate affinity estimation and more reliable affinity ranking. Collectively, these findings highlight the robustness and predictive strength of DTBind in quantitative drug–target affinity prediction.

To examine its generalization ability across diverse protein classes, we partitioned the test set into 6 representative families—kinases, nuclear receptors, ion channels, immunoglobulins, serine proteases, and others—and evaluated its performance per family (Fig. 4F and G; with detailed metrics provided in Tables S5 and S6). After evaluation, DTBind still achieved higher performance than all compared baseline models across most protein families. For instance, Pearson/RMSE reached 0.9132/0.8509 for immunoglobulins, 0.9583/0.9251 for serine proteases, and 0.7299/1.0471 for kinases. In contrast, baselines displayed substantial variability, with some yielding negative correlations in certain families. These results indicate that DTBind captures distinct interaction patterns across different protein families while maintaining stable performance on structurally and functionally diverse targets.

In conclusion, these results show that DTBind achieves more accurate and reliable affinity prediction than baseline models across both the full dataset and diverse protein families. A likely reason for the weaker performance of baselines is that they do not explicitly incorporate complex structural information, thereby missing critical geometric cues for drug–target interactions. In contrast, DTBind represents each complex as a residue–atom heterogeneous graph enriched with geometric relationships, providing a structured view of potential interaction patterns.

### Determinant-aware encodings and adaptive decodings enhance molecular recognition

We conducted systematic ablation experiments to dissect the contributions of DTBind’s major components across 3 dimensions of molecular recognition. Specifically, we selectively ablated determinant-aware protein encodings and key components of the information-adaptive decoders to assess their contributions to predictive performance.

**Fig. 5.**
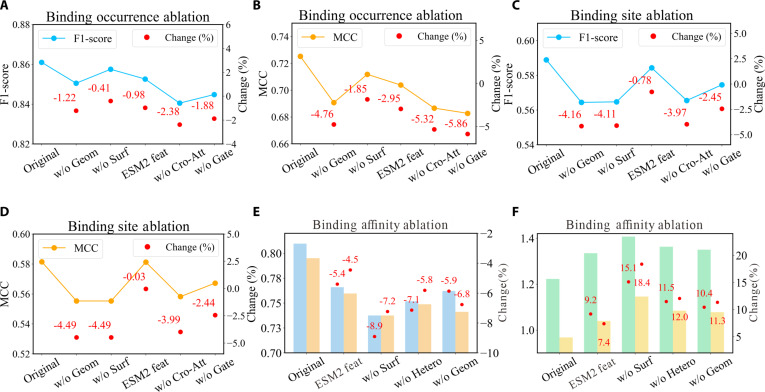
Ablation study of DTBind. (A and B) Ablation results for binding occurrence prediction, showing metric variations in F1-score (A) and MCC (B). (C and D) Ablation results for binding site prediction, showing metric variations in F1-score (C) and MCC (D). Curves represent metric changes, with red dots indicating relative performance drops. (E and F) Ablation results for binding affinity prediction: (E) reports Pearson (blue) and Spearman (orange) correlations, with red dots denoting relative percentage decreases; (F) reports RMSE (green) and MAE (yellow), with red dots denoting relative percentage increases.

In binding occurrence prediction, removing geometric edges (w/o Geom) or surface cues (w/o Surf) reduces F1-score by 1.22%/0.41% and MCC by 4.67%/1.85% (Fig. 5A and B, with detailed metrics provided in Table S7). In binding site localization, eliminating them lowers F1-score by 4.11% to 4.16% and MCC by 4.49% (Fig. 5C and D, with detailed metrics provided in Table S8). In affinity estimation, the effects are more pronounced: Excluding surface features increases RMSE by 15.1% and MAE by 18.4%, while removing geometric edges decreases Pearson correlation by 5.9% (Fig. 5E and F, detailed metrics in Table S9). To comprehensively assess the influence of pretrained protein representations across all predictive tasks, we systematically replaced ProtT5 [53] features with ESM2 [54] features in the binding occurrence, binding site, and affinity prediction modules (Tables S7 to S9). The results reveal consistent performance trends across the 3 tasks. For binding occurrence prediction, replacing ProtT5 with ESM2 led to small declines in F1 (−2.95%) and MCC (−0.98%). For binding site prediction, performance remained nearly equivalent, with F1 (−0.78%) and MCC (−0.03%) differing by less than 1%. In contrast, affinity prediction exhibited more substantial deviations, with a 9.2% increase in RMSE and a 5.4% decrease in Pearson correlation. These observations suggest that while ProtT5 offers a modest advantage in affinity estimation, both pretrained models provide comparably expressive embeddings for binding occurrence and residue-level site recognition tasks. Collectively, the results underscore that the synergy between sequence semantics, geometric encoding, and surface-aware representation plays a decisive role in achieving high predictive accuracy across all modules of DTBind.

Beyond these determinant-based protein representations, information-adaptive decodings further enhance their predictive accuracy. For binding occurrence, simplifying dual-channel MCA to a single head (w/o Cro-Att) decreases F1-score by 2.38% and MCC by 5.32%, while removing the bilateral gating mechanism (w/o Gate) causes an additional 5.86% drop in MCC, confirming their indispensable roles in stabilizing global feature alignment. For binding site localization, reducing atom–residue interaction mechanism to a single attention head (w/o Cro-Att) decreases F1-score by 3.97% and MCC by 3.98%, underscoring the necessity of multi-channel local exchange. For affinity estimation, removing the pocket-centered atom–residue heterogeneous graph networks (w/o Hetero) lowers Pearson and Spearman correlations by 7.1% and 5.8%, respectively.

Collectively, these results demonstrate that DTBind’s superior performance derives from the joint use of determinant-aware encoders and information-adaptive decoders. Accurate encoding of protein sequence, geometry, and surface chemistry provides a determinant-based foundation, while information-adaptive decoders highlight features most relevant to corresponding tasks.

### Mechanism-based protein encodings gradually drive accurate predictions

Accurate identification of drug–target molecular recognition requires full consideration and proper encoding of key determinants that govern molecular recognition, including shape complementarity, functional alignment, physicochemical compatibility, and surface exposure. To assess the interpretability and functional relevance of these determinant embeddings in DTBind, we applied residue-level Grad-CAM [55] analysis across all samples in the binding site prediction test set (analysis details in Note S1). Grad-CAM assigns each residue an activation score, with larger scores indicating higher contributions to the predicted binding probability.

We evaluated the Grad-CAM activation scores at 3 feature-integration stages that correspond to progressively richer determinant encodings: stage 1 (node feature): pretrained sequence features encoding residue-level physicochemical and functional properties; stage 2 (node + edge feature): addition of geometric edge features capturing inter-residue spatial arrangement and local topology; stage 3 (node + edge + surface feature): further integration of residue-level surface features reflecting pocket morphology and accessibility. These 3 stages successively introduce 3 key determinants of molecular recognition, allowing to examine how each determinant enhances the model’s discriminative capacity.

**Fig. 6.**
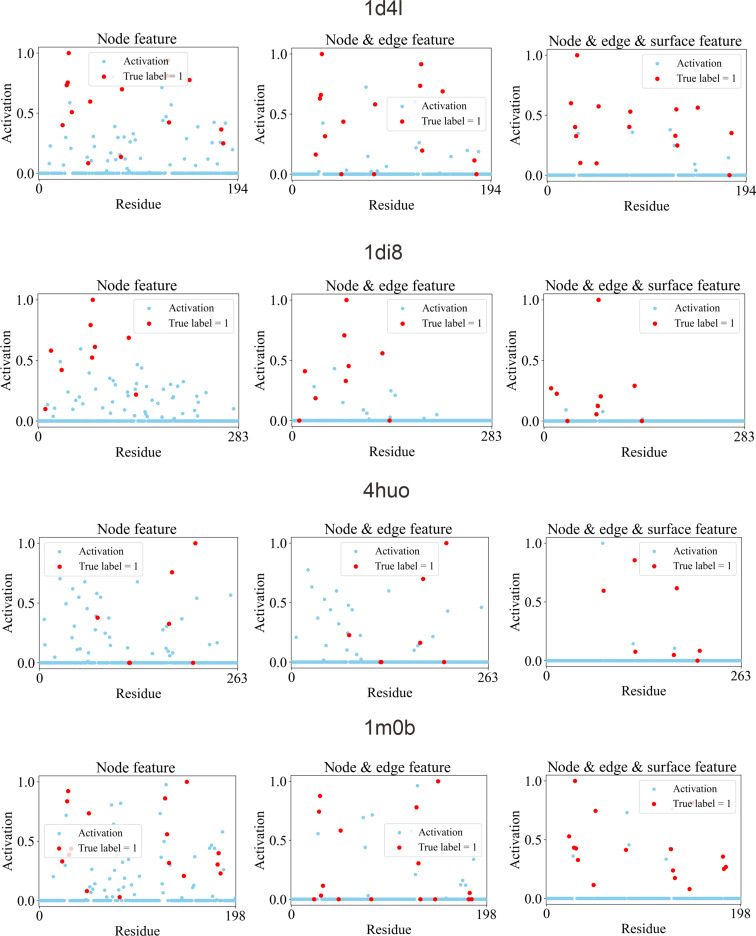
Residue-level Grad-CAM explains the roles of 3 mechanism-based features. Hierarchical encoding visualizations for 4 representative drug–target pairs (PDB IDs: 1d4l, 1di8, 4huo, and 1m0b) across 3 stages of feature encoding: node features (left), node embeddings plus geometric edge features (middle), and further fused surface features (right). Each scatterplot shows residue indices (*x* axis) versus normalized activation scores (*y* axis), with red points representing true binding residues and blue indicating nonbinding residues.

To quantitatively explore the correspondence between Grad-CAM activation scores and annotated binding residues, we performed an enrichment of residues with high activation scores among known binding sites. In detail, we measured the fraction of true binding residues among top 5% residues ranked by Grad-CAM activation scores and reported fold enrichment relative to the background binding ratio (~2.8%). The results show a clear progressive improvement in alignment between Grad-CAM activation scores and true binding residues, with top 5% binding ratio increasing from 0.274 (stage 1) to 0.275 (stage 2) and to 0.340 (stage 3), corresponding to a fold enrichment rising from 10.91× (stage 1) to 10.99× (stage 2) and to 12.94× (stage 3). For visual confirmation, Fig. 6 illustrates Grad-CAM heatmaps for representative complexes [Protein Data Bank (PDB) IDs: 1d4l, 1di8, 4huo, and 1m0b] across the 3 stages (from left to right). With only sequence embeddings, activation scores are diffuse and extend beyond true pockets. Adding geometric features refines localization, emphasizing residues forming the pocket’s structural boundary. The incorporation of surface features further sharpens this focus, restricting high activation scores almost exclusively to experimentally verified binding residues.

Collectively, the quantitative and qualitative analyses demonstrate that DTBind’s hierarchical encoding progressively integrates the essential determinants of molecular recognition. Each encoding stage contributes a distinct but indispensable component. Their stepwise integration systematically enhances the model’s ability to distinguish binding from nonbinding residues.

### DTBind provides consistent molecular recognition predictions across diverse complexes

**Fig. 7.**
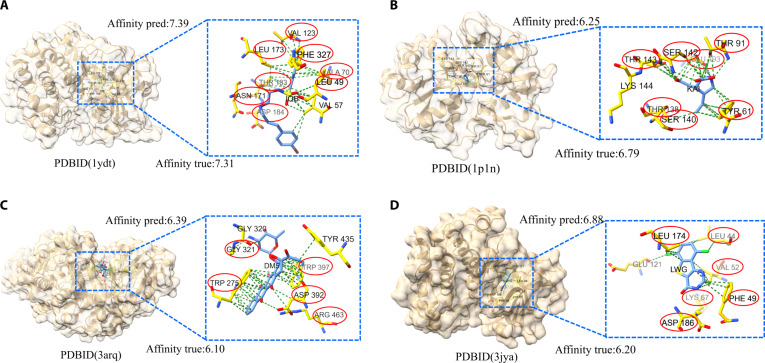
DTBind’s hierarchical predictions across diverse complexes. For each complex [(A), 1ydt; (B), 1p1n; (C), 3arq; (D), 3jya], the left panel displays the overall protein–ligand structure, and the right panel provides a close-up view of the binding region. The ligand is shown as blue sticks, ground-truth binding residues identified by PLIP [60] are shown as yellow sticks, and the predicted binding residues by DTBind are highlighted with red halos.

To evaluate the practical utility of DTBind in comprehensive molecular recognition, we examined 4 representative complexes spanning diverse biological systems and functional classes (PDB IDs: 1ydt [56], 1p1n [57], 3arq [58], and 3jya [59]; Fig. 7). These examples include kinase inhibition (1ydt [56] and 3jya [59]), neurotransmitter receptor modulation (1p1n [57]), and enzyme inhibition (3arq [58]), thereby covering structurally and functionally heterogeneous drug–target interactions. For each complex, DTBind simultaneously generated 3 levels of predictions: (a) binary interaction classification to determine whether binding occurs, (b) residue-level binding site localization to identify specific amino acids involved in the interaction, and (c) quantitative affinity estimation to reflect binding strength. DTBind produced consistent and mutually coherent outputs across all 4 cases, together constructing a multi-level view of drug–target molecular recognition.

Across the 4 cases, DTBind consistently predicted the correct binding outcome, demonstrating reliable binary recognition. The residue-level predictions largely matched ground-truth annotations derived from PLIP (Protein–Ligand Interaction Profiler) [60] analyses, with typically only 1 or 2 binding residues missed per complex, highlighting the model’s precision in localizing the functional interface. For example, in 1ydt [56] (PKA-Cα with H89), DTBind identified nearly all key residues in the adenosine triphosphate (ATP)-binding cleft, correctly reflecting interactions with the hinge region that regulates phosphorylation. In 1p1n [57] (GluR2 ligand-binding domain with kainate), the predicted residues closely recapitulated the orthosteric clamshell pocket that mediates excitatory neurotransmission. In 3arq [58] (GH18 chitinase A with idarubicin), critical catalytic groove residues were accurately identified, and in 3jya [59] (Pim-1 kinase with LWG), the ATP hinge residues essential for catalytic activity were largely recovered. These results demonstrate that DTBind precisely recognizes binding sites, establishing a foundation for rational drug design and for elucidating the mechanistic principles of molecular recognition. In parallel, DTBind’s affinity predictions closely matched experimental values across all 4 complexes, with errors within 0.7 units, underscoring the model’s ability to quantify interaction strength. Collectively, these case studies show that DTBind can provide a relatively comprehensive description of drug–target molecular recognition, facilitating the evaluation of binding stability and affinity.

### DTBind provides reliable predictions for proteins without experimental structures

DTBind was also applied to exploring drug–target binding regions on 3 drug–target pairs without experimental complex structures that were randomly selected from the BindingDB [61] database to evaluate whether DTBind can reliably predict binding sites for proteins whose experimentally resolved structures are unavailable. In detail, the protein with UniProt [62] ID B0BL08 is a bacterial dihydrofolate reductase (DHFR), a key enzyme in folate metabolism and an established antimicrobial drug target. Its associated ligand (CID 13054042) is a pyrimidine-2,4-diamine derivative, structurally related to trimethoprim-like inhibitors designed to block bacterial DHFR activity. The protein with UniProt [62] ID P48729 is casein kinase I isoform α (CSNK1A1), a serine/threonine protein kinase that regulates Wnt signaling, circadian rhythm, and DNA repair. The ligand (CID 71455731) belongs to a heteroaryl pyrimidinone scaffold, consistent with ATP-competitive kinase inhibitors developed to modulate dysregulated phosphorylation in cancer and inflammatory diseases. The protein with UniProt [62] ID Q15059 is bromodomain-containing protein 3 (BRD3), a member of the bromodomain and extra-terminal motif (BET) family that recognizes acetylated lysine residues on histones and regulates transcriptional programs linked to cancer and inflammation. The ligand (CID 91826377) is a triazolopyridine-based BET inhibitor, designed to occupy the acetyl-lysine recognition pocket of BRD3 and disrupt oncogenic transcriptional machinery.

**Fig. 8.**
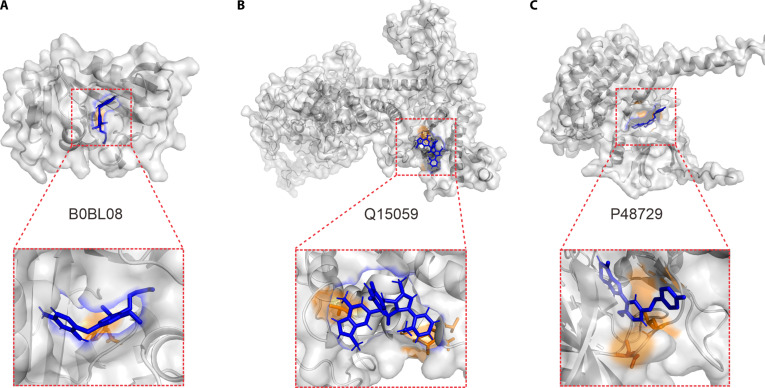
Extension of DTBind to proteins lacking experimental structures. Representative drug–target pairs [(A), B0BL08; (B), P48729; (C), Q15059] were selected from the BindingDB [61] database, with protein structures obtained from AlphaFoldDB [63]. Drugs are shown as blue sticks. The predicted binding residues are displayed as orange sticks, with their corresponding surface regions highlighted in orange. Molecular dynamics (MD) simulations further confirmed that these predicted sites spatially overlap with ligand-accessible cavities.

The predicted protein structures were retrieved from the AlphaFold Protein Structure Database [63], and residue-level binding sites were generated by molecular docking and MD simulations [64] to provide a reliable physical validation (with method of validation details provided in Note S2). Across all the 3 cases, DTBind consistently predicted residue binding sites that overlapped with ligand-accessible pockets identified by MD simulations. Strikingly, the predicted binding residues did not scatter across the protein surface but instead converged onto spatially contiguous regions that corresponded to dynamically stable cavities. In the 3 representative proteins (UniProt [62] IDs: B0BL08, P48729, and Q15059), the predicted sites and its surfaces (highlighted in orange) coincided with the binding pockets explored by ligands (blue) during MD trajectories (Fig. 8). This concordance underscores that DTBind can not only generalize beyond benchmark datasets but also produce predictions that are physically consistent with structural dynamics. These results highlight the practical utility of DTBind for drug discovery applications.

## Discussion

In this study, we introduce DTBind, a mechanism-driven deep learning framework that reformulates drug–target interaction prediction as a hierarchical molecular recognition problem encompassing 3 interrelated predictive levels—binding occurrence prediction, binding site localization, and binding affinity estimation. By coupling a shared determinant-aware encoder with information-adaptive decoders that progressively leverage sequence-, structure-, and complex-level cues, DTBind hierarchically exploits all available data modalities to model drug–target molecular recognition in a coherent and mechanistically meaningful manner. Comprehensive evaluations show that DTBind consistently outperforms state-of-the-art baselines across diverse benchmarks. Moreover, for drug–target pairs without experimental structures, predicted binding sites validated by MD simulations further support the robustness and real-world applicability of the framework.

The performance advantages of DTBind arise from 2 complementary design principles. First, shared and determinant-aware protein encodings capture essential factors of molecular recognition, including sequence-derived physicochemical properties, local geometric relationships among residues, and surface accessibility. The progressive incorporation of these features improves predictive accuracy across all 3 levels of molecular recognition—binding occurrence, binding site, and binding affinity. Second, the information-adaptive decoders align model predictions with task-specific requirements: global intermolecular representations for interaction occurrence, residue-level distinctions for binding sites, and heterogeneous atom–residue interactions for affinity estimation. The synergy between these encoders and decoders enables DTBind to move beyond fragmented prediction and provide a coherent, multi-level description of molecular recognition.

Despite these advances, several limitations should be noted. First, the availability of high-quality drug–target complex structures remains limited, constraining training coverage and potentially affecting predictions for under-characterized proteins. Second, predictions based on computationally derived structures, such as those from AlphaFold2 [65], may be less reliable for intrinsically disordered or locally folded regions. Third, constructing heterogeneous protein–drug graphs and training large-scale models incur considerable computational costs, which may hinder ultra-high-throughput applications. These challenges underscore the need for the continued integration of emerging structural prediction methods, larger curated datasets, and efficient model architectures to enhance both scalability and reliability.

Beyond the immediate scope of drug–target interactions, the principles underlying DTBind—determinant-aware encoding and multi-level, information-adaptive decoding—may extend to other biomolecular recognition problems [66–70], including protein–protein, protein–RNA, and antibody–antigen interactions. By providing open-access codes, pretrained models, and processed datasets, DTBind contributes a practical tool for rational drug design and repurposing. Taken together, DTBind demonstrates how mechanism-driven deep learning can advance the systematic understanding of molecular recognition while remaining applicable under realistic constraints of early-stage drug discovery.

## Methods

### Data preparation

We compiled datasets from multiple publicly available resources, including PDBbind (v2020) [47], PDB [71], BioSNAP [44], PubChem [72], and AlphaFoldDB [63], to support different predictions in our study.

#### Binding occurrence prediction

For the binding occurrence prediction, we adopted the BioSNAP [44] dataset following the construction and splitting protocol of MolTrans [42]. The dataset comprises 4,510 unique compounds and 2,181 protein targets, with experimentally validated positive interactions paired with an equal number of negative samples generated from previously unseen compound–protein pairs. For determining binding occurrence, rather than mechanistic details, global sequence-derived physicochemical properties and folding constraints are sufficient, while predicted protein structures supply the required structural context. Structural models were retrieved from AlphaFoldDB [63] based on UniProt [62] identifiers, and for proteins absent from AlphaFoldDB [63], models were generated using Chai-1 [73]. Due to input length limitations of Chai-1 [73], extremely long sequences could not be modeled; accordingly, drug–target pairs lacking valid structures were excluded. The resulting dataset contains 13,826 positive and 13,647 negative interactions, split into 19,088, 2,720, and 5,453 pairs for training, validation, and testing, respectively, following the 7:1:2 protocol of MolTrans [42].

#### Binding site prediction

For the binding site prediction, we leveraged the PDBbind (v2020) [47] database, which provides high-quality protein–ligand complex structures. Binding site annotations were generated using PLIP [60], which identifies 7 categories of noncovalent interactions—hydrogen bonds, hydrophobic contacts, π–π stacking, π–cation interactions, salt bridges, water bridges, and halogen bonds—based on atomic types, interatomic distances, and bond geometries. Complexes for which PLIP [60] failed to generate valid interaction annotations were removed, as were ligands that could not be processed by RDKit [74]. The curated dataset was partitioned into training, validation, and test sets at a 7:1:2 ratio, comprising 9,760, 1,384, and 2,776 protein–ligand complexes, respectively. At the residue level, the final dataset contains 134,906 binding site residues versus 6,489,442 nonbinding residues, yielding a binding-to-nonbinding ratio of approximately 1:48. This pronounced imbalance reflects the inherent sparsity of functional binding sites on protein surfaces and underscores the necessity of models that are robust to highly skewed label distributions.

#### Binding affinity prediction

For the affinity prediction, we again used PDBbind (v2020) [47], which provides experimentally measured binding affinities [inhibition constant (*K*_i_), dissociation constant (*K*_d_), and median inhibitory concentration (IC_50_)] along with corresponding 3D structures. To ensure reliable data quality, we applied stringent filtering criteria: (a) affinities must be reported as exact numerical values rather than ranges or approximations; (b) ligands must be processable by RDKit [74]; and (c) at least one noncovalent interaction must be detected by PLIP [60]. After filtering, 14,785 compound–protein pairs were retained. To facilitate model training, affinity values were transformed into p-affinity scores defined as

$$p\left(\text{affinity}\right)=-{\text{log}}_{10}\left(\text{affinity}/\left[M\right]\right)$$ (1)

ensuring consistency of scale and interpretability. Following the standard evaluation protocol, the CASF-2016 benchmark set [52] (285 complexes) was held out entirely as the test set, while the remaining general set was split into training and validation subsets at a 7:1 ratio. For this prediction, the protein pocket structures from PDBbind [47] and the corresponding protein–ligand complex conformations were further utilized to construct heterogeneous molecular graphs, serving as inputs for affinity estimation.

### The architecture of DTBind

DTBind employs a unified framework to model protein-drug molecular recognition. It predicts binding occurrence using predicted structures from sequences and drug structures, localizes binding sites with experimental protein structures, and estimates binding affinity when complex structures are available. In all cases, DTBind converts structural inputs into determinant-aware representations that preserve structural and physicochemical information, which are then refined by graph-based feature encoders. Task-specific, adaptive decoders finally generate the predictions, capturing multiple aspects of molecular recognition. The framework is detailed next from 3 perspectives: molecular representation, graph-based feature encoders, and information-adaptive decoders.

#### Molecular representation

DTBind constructs graph-based protein representations that preserve residue-level spatial relationships, and surface-based representations that reflect the overall geometric and physicochemical properties of the protein surface. For drugs, it builds graph representations to model atomic composition and connectivity. Together, these multimodal representations provide a solid foundation for downstream feature encoding and decoding.

For binding affinity prediction, we use the pocket structures provided by the PDBbind database, which provides curated protein–ligand complexes along with the corresponding pocket PDB files for each complex. These pocket files include not only the residues that directly constitute the ligand-binding region but also the neighboring residues that contribute to the overall pocket geometry, ensuring structural consistency and biological relevance.

##### Protein and pocket representation

To represent a protein or a pocket of length $N_{p}$, we constructed a residue-level directed graph $G_{p}=\left(V_{p},\;E_{p}\right)$, where nodes $V_{p}$ correspond to amino acid residues and edges $E_{p}$ connect residues within a 12-Å cutoff distance (measured between α-carbon atoms).

To ensure that the graph representation retained the principal determinants of molecular recognition, we assigned complementary features to its nodes and edges. For each node i, we apply the protein large language model ProtT5 to generate an embedding vector $v_{i}^{p}\in R^{1024}$ that serves as its node feature, capturing the underlying physicochemical properties and functional context of the residue. Edge features were defined by rotation-invariant geometric descriptors. The local, intrinsic orthonormal coordinate frame was established for each residue (see Note S3 for construction details). For a connected residue pair $\left(i,j\right)$, the edge feature vector $e_{\mathrm{ij}}^{p}$ was computed as:

$$e_{\mathrm{ij}}^{p}=\left[x_{\mathrm{ij}},\;y_{\mathrm{ij}},\;z_{\mathrm{ij}},\;\phi \left(d_{\mathrm{ij}}\right),\;q_{\mathrm{ij}}\right]\in R^{8}$$ (2)

Here, $\left(x_{\mathrm{ij}},\;y_{\mathrm{ij}},\;z_{\mathrm{ij}}\right)$ are the local coordinates of residue j’s α-carbon in residue i’s frame. $\phi \left(d_{\mathrm{ij}}\right)=\text{exp}\left(-\frac{d_{\mathrm{ij}}^{2}}{2\sigma^{2}}\right)$ (σ=1.0) is the radial basis function encoding the inter-residue distance $d_{\mathrm{ij}}$. $q_{\mathrm{ij}}\in R^{4}$ is the unit quaternion representing the rotation between the local frames of residue i and j. T represents the transpose operation.

In addition to the protein graph representation, we further derive residue-level surface descriptors to capture complementary cues from the solvent-exposed regions of the protein (details in Note S6). For each residue i, we compute a 10D vector $s_{i}$, where the first 4 dimensions encode geometric shape descriptors (mean curvature, Gaussian curvature, shape index, and curvedness) averaged over assigned surface vertices, and the remaining 6 dimensions encode the residue’s chemical environment via the one-hot atom-type composition (C, H, O, N, S, other) averaged over all atoms of residue. These features capture critical determinants of molecular recognition: The geometric terms delineate pocket accessibility and morphology, while the chemical terms characterize the local physicochemical environment of the exposed surface. The collection of all residues forms a surface tensor $S={\left[s_{1},\ldots ,s_{N}\right]}^{T},s_{i}\in R^{10}$, which was fused downstream to enrich recognition of ligand-accessible pockets.

##### Drug representation

Each molecular entity (including drugs and small molecules) with an atomic cardinality of $N_{d}$ is processed with RDKit and represented as an undirected molecular graph $G_{d}=\left(V_{d},\;E_{d}\right)$, where $V_{d}$ denotes the set of atoms and $E_{d}$ denotes the set of chemical bonds. Each atom node i is associated with a node feature vector $v_{i}^{d}\in R^{82}$, which encodes atom-specific properties including atom type, degree, explicit valence, implicit valence, and aromaticity using one-hot representations. Similarly, each edge $\left(i,\;j\right)\in E_{d}$ is associated with an edge feature vector $e_{\mathrm{ij}}^{d}\in R^{6}$, which concatenates one-hot encodings of bond type (single, double, triple, or aromatic) with additional indicators for conjugation and ring membership. This representation provides a rich, atom-level structural encoding suitable for graph-based learning.

#### Graph-based feature encoders

##### Protein feature encoder

DTBind builds a multi-layer graph convolution network (GCN) on protein graph representations to extract deep protein features and integrates them with surface features to obtain the final protein representation. The node features ${\left\{v_{i}^{p}\right\}}_{i=1}^{N_{p}}$ and surface features ${\left\{s_{i}\right\}}_{i=1}^{N_{p}}$ are first projected into a hidden space for initialization:

$$h_{i}^{0}=\sigma \left(\mathrm{FC}\left(v_{i}^{p}\right)\right)\in R^{h_{1}}$$ (3)

$$s_{i}^{\prime }=\sigma \left(\mathrm{FC}\left(s_{i}\right)\right)\in R^{h_{2}}$$ (4)

where $\mathrm{FC}\left(\cdot \right)$ is fully connected layer and $\sigma \left(\cdot \right)$ is LeakyRELU activation function.

At layer t, node i aggregates information from its neighboring $j\in N\left(i\right)$. This information is determined jointly by the features of node j, the attributes of the edge connecting i and j, and the attention coefficient $\alpha_{\mathrm{ij}}$:

$$m_{\mathrm{ij}}^{t}=\alpha_{\mathrm{ij}}^{t}\left(W_{v}h_{j}^{t}+W_{e}e_{\mathrm{ij}}^{p}\right)$$ (5)

where $W_{v}\in R^{h_{1}\times h_{1}}$ and $W_{e}\in R^{h_{1}\times h_{1}}$ are learnable parameter matrices. The attention coefficient $\alpha_{\mathrm{ij}}$ is computed from both node–node and edge–edge similarity:

$$\alpha_{\mathrm{ij}}^{t}=\lambda \alpha_{v}^{\mathrm{ij},t}+\left(1-\lambda \right)\alpha_{e}^{\mathrm{ij},t}$$ (6)

$$\alpha_{v}^{\mathrm{ij},t}=\text{Softmax}\left(\frac{{\left(W_{Q}^{v}h_{j}^{t}\right)}^{⊤}\left(W_{K}^{v}h_{i}^{t}\right)}{\sqrt{d_{h}}}\right)$$ (7)

$$\alpha_{e}^{\mathrm{ij},t}=\text{Softmax}\left(\frac{{\left(W_{Q}^{e}e_{\mathrm{ij}}^{p}\right)}^{⊤}\left(W_{K}^{e}e_{\mathrm{ij}}^{p}\right)}{\sqrt{d_{e}}}\right)$$ (8)

where $W_{Q}^{v},W_{K}^{v}\in R^{d_{h}\times d_{h}},W_{Q}^{e},W_{K}^{e}\in R^{d_{e}\times d_{e}}$ are learnable parameter matrices, and both $d_{h}$ and $d_{e}$ represent hidden layer dimensions. In this work, λ is set to 0.5.

We aggregate information from all neighboring nodes to node i while incorporating a residual connection to preserve the original information:

$$h_{i}^{t+1}=\sigma \left(W_{\text{out}}h_{i}^{t}+\sum_{j\in N\left(i\right)}m_{\mathrm{ij}}^{t}\right)+h_{i}^{t}$$ (9)

where $W_{\text{out}}\in R^{h_{1}\times h_{1}}$ is a learnable parameter matrix.

After T (is set to 3 in this work) layers, we obtain residue-level protein embeddings $H_{p}^{\prime }={\left[h_{1}^{T},\cdots h_{N_{p}}^{T}\right]}^{T}\in R^{N_{p}\times h_{1}}$. Subsequently, it is concatenated with the surface features $S^{\prime }={\left[s_{1}^{\prime },\ldots ,\;s_{N_{p}}^{\prime }\right]}^{T}\in R^{N_{p}\times h_{2}}$, and after processing through a multi-layer perceptron (MLP), the final protein encoding is obtained:

$$H_{p}^{\text{final}}=\mathrm{MLP}\left(\left[H_{p}^{\prime }\;\|\;S^{\prime }\right]\right)\in R^{N_{p}\times h_{1}}$$ (10)

where ‖ denotes concatenation along the feature dimension.

##### Drug feature encoder

DTBind adopts a message-passing neural network (MPNN) with 3 layers on the drug graph. The node features ${\left\{v_{i}^{d}\right\}}_{i=1}^{N_{d}}$ are first projected into a hidden space for initialization:

$$h_{i}^{0}=\sigma \left(\mathrm{FC}\left(v_{i}^{d}\right)\right)$$ (11)

For an atom i at layer t, a message forming its neighbor j is:

$$m_{\mathrm{ij}}^{t}=\sigma \left(W_{u}\left[h_{j}^{t}\|e_{\mathrm{ij}}^{d}\right]\right)$$ (12)

where $W_{u}\in R^{h_{3}\times \left(h_{3}+6\right)}$ is a learnable parameter matrix.

The atom representation is updated by concatenating its previous state with the sum of incoming messages, followed by a linear transformation:

$$h_{i}^{t+1}=W_{C}\left[\;h_{i}^{t}\|\sum_{j\in N\left(i\right)}m_{\mathrm{ij}}^{t}\;\right]$$ (13)

where $W_{C}\in R^{h_{3}\times 2h_{3}}$ is a learnable parameter matrix. After passing through T layers of MPNN, we obtain the final drug encoding $H_{d}^{\text{final}}={\left[h_{1}^{T},\cdots h_{N_{d}}^{T}\right]}^{T}\in R^{N_{d}\times h_{3}}$.

#### Information-adaptive decoders

##### Binding occurrence decoder

This decoder predicts the probability of binding. The encoded protein and drug features, $H_{p}^{\text{final}}$and $H_{d}^{\text{final}}$, are first projected:

$$H_{p}=\sigma \left(H_{p}^{\text{final}}W_{p1}\right)$$ (14)

$$H_{d}=\sigma \left(H_{d}^{\text{final}}W_{d1}\right)$$ (15)

where $W_{p1}\in R^{h_{4}\times h_{4}},W_{d1}\in R^{h_{4}\times h_{4}}$ are learnable parameter matrices.

A dual-pathway cross-attention mechanism then refines these representations. Protein features are updated using protein as query and drugs as keys/values, and vice versa:

$$H_{p}^{\prime }=\mathrm{LN}\left(H_{p}+\mathrm{MCA}\left(H_{p}W_{Q}^{p},\;H_{d}W_{K}^{d},\;H_{d}W_{V}^{d}\right)\right)$$ (16)

$$H_{d}^{\prime }=\mathrm{LN}\left(H_{d}+\mathrm{MCA}\left(H_{d}W_{Q}^{d},\;H_{p}W_{K}^{p},\;H_{p}W_{V}^{p}\right)\right)$$ (17)

where $W_{Q}^{p},W_{K}^{d},W_{V}^{d},W_{Q}^{d},W_{K}^{p},W_{K}^{p}\in R^{h_{4}\times h_{4}}$ are learnable parameter matrices and MCA denotes multi-head cross-attention mechanism that allows the model to simultaneously focus on different aspects of the protein and drug features by utilizing multiple attention heads. Detailed equations are provided in Note S4.

An adaptive gating mechanism fuses the original and attended features:

$$H_{\mathrm{pf}}=g_{p}⊙H_{p}^{\prime }+\left(1-g_{p}\right)⊙H_{p},g_{p}=\sigma \left(\left[H_{p}^{\prime }∥H_{p}\right]W_{p}\right)$$ (18)

$$H_{\mathrm{df}}=g_{d}⊙H_{d}^{\prime }+\left(1-g_{d}\right)⊙H_{d},g_{d}=\sigma \left(\left[H_{d}^{\prime }∥H_{d}\right]W_{d}\right)$$ (19)

where $W_{p},W_{d}\in R^{2h_{4}\times h_{4}}$ are learnable parameter matrices and ⊙ denotes element-wise multiplication.

For both modalities, to preserving original global-level information while enabling nonlinear feature transformation, we aggregate the features of all residues in the protein (or all atoms in the drug) via mean and max pooling, then refine using residual MLPs:

$$z_{p}=\text{Mean}\left(H_{\mathrm{pf}}\right)+\text{Max}\left(H_{\mathrm{pf}}\right)$$ (20)

$$z_{d}=\text{Mean}\left(H_{\mathrm{df}}\right)+\text{Max}\left(H_{\mathrm{df}}\right)$$ (21)

$$z_{p}^{\prime }=\mathrm{MLP}\left(z_{p}\right)+W_{p}^{r}z_{p}$$ (22)

$$z_{d}^{\prime }=\mathrm{MLP}\left(z_{d}\right)+W_{d}^{r}z_{d}$$ (23)

where $W_{p}^{r},W_{d}^{r}\in R^{h_{4}\times h_{4}}$ are learnable parameter matrices.

Finally, the joint interaction representation is constructed as

$$z=z_{p}^{\prime }∥z_{d}^{\prime }∥\left(z_{p}^{\prime }⊙z_{d}^{\prime }\right)∥\left(z_{p}^{\prime }-z_{d}^{\prime }\right)\in R^{4h_{4}}$$ (24)

and the binding probability is predicted via a 4-layer MLP classifier:

$$\hat{y}=\text{Classification}\left(z\right)$$ (25)

where $\text{Classification}\left(\cdot \right)$ consists of 4 fully connected layers, interleaved with nonlinear activations, LayerNorm, and a dropout layer to improve generalization. The feature dimensions are progressively reduced from $4h_{4}\to 2h_{4}\to h_{4}\to h_{4}/2\to 1$. The final output is passed through a softmax function to produce the classification probabilities.

##### Binding site decoder

This decoder identifies binding residues at the residue level. The encoded protein and drug features, $H_{p}^{\text{final}}$ and $H_{d}^{\text{final}}$, are first projected:

$$H_{p}=\sigma \left(H_{p}^{\text{final}}W_{p2}\right)$$ (26)

$$H_{d}=\sigma \left(H_{d}^{\text{final}}W_{d2}\right)$$ (27)

where $W_{p2},W_{d2}\in R^{h_{5}\times h_{5}}$ are learnable parameter matrices.

To better capture the global interaction information for protein binding site prediction, we apply 2 consecutive multi-head attention modules. In the first module, protein features serve as queries, while drug features are used as keys and values to update the protein representation. This is followed by a self-attention module that further refines the protein representation:

$$H_{c}=H_{p}+\mathrm{MCA}\left(H_{p}W_{Q}^{p},\;H_{d}W_{K}^{d},\;H_{d}W_{V}^{d}\right)$$ (28)

$$H_{s}=\mathrm{MSA}\left(H_{c}W_{Q},\;H_{c}W_{K},\;H_{c}W_{V}\right)$$ (29)

where $W_{Q}^{p},W_{K}^{d},W_{V}^{d},W_{Q},W_{K},W_{V}\in R^{h_{5}\times h_{5}}$ are learnable parameter matrices and the detailed equations of MCA and multi-head self-attention (MSA) are provided in Note S4.

The outputs are fused via adaptive gating:

$$Z=g⊙H_{c}+\left(1-g\right)⊙H_{s}$$ (30)

$$g=\sigma \left(\left[H_{c}\;\|\;H_{s}\right]W_{g}\right)$$ (31)

where $W_{g}\in R^{2h_{5}\times h_{5}}$ is a learnable parameter matrix.

Finally, the binding probability for residue i is given by:

$$\hat{y_{i}}=\text{sigmoid}\left(W_{r}z_{i}+\mathrm{MLP}\left(h_{f,i}\right)\right)$$ (32)

where $z_{i}$ is the fused embedding for residue i and the MLP consists of 2 linear layers transforming the hidden dimension from $h_{5}\to 2h_{5}\to 1$. $W_{r}\in R^{1\times h_{5}}$ is a parameter matrix.

Because the binding site prediction task involves a highly imbalanced class distribution, we employed the focal loss to mitigate this issue and improve model sensitivity to minority classes. Detailed formulation and parameter settings are provided in Note S7.

##### Binding affinity decoder

To estimate binding affinity, we construct a directed heterogeneous graph $G_{h}=\left(V_{p},\;V_{d},\;E_{p\to d},\;E_{d\to p}\right)$ that explicitly models the critical intermolecular interface. Here, $V_{p}$ denotes pocket residues and $V_{d}$ denotes drug atoms. To explicitly model the critical intermolecular interface, which is the primary contributor to binding affinity, the heterogeneous graph was constructed centered on the protein pocket. The protein nodes in this heterogeneous graph correspond exclusively to the pocket residues defined in the molecular representation stage. This design focuses the model on the region where key biophysical determinants, such as shape complementarity and functional group matching, are most pronounced.

To capture bidirectional interaction contexts, we create directed edges in both directions. For each residue $i\in V_{p}$, we connect it to its k=6 nearest drug atoms $j\in V_{d}$, forming edges $E_{d\to p}$ (information flow from drug to protein). Similarly, for each drug atom $j\in V_{d}$, we connect it to its k=6 nearest residues $i\in V_{p}$, forming edges $E_{p\to d}$. The 6 nearest neighbors are determined based on Euclidean distances in 3D space, ensuring that only spatially proximal atoms or residues contribute to the interaction modeling. Node features are initialized from the respective encoder outputs: $H_{\text{pocket}}^{\text{final}}$ for residues and $H_{d}^{\text{final}}$ for drug atoms, where $H_{\text{pocket}}^{\text{final}}=\left\{h_{i}\in H_{p}^{\text{final}}|i\in \text{pocket}\right\}$. Edge features encode the precise 3D spatial relationship between residues and atoms.

In constructing edge features, we fully incorporate the topological information reflecting the relative orientation and distance between the connected residue and atom. Specifically, we first establish a local coordinate system. For each protein residue, the α-carbon atom is set as the origin. The *z* axis is aligned with the vector pointing toward its nearest drug atom, and the *xz* plane is defined by the vector connecting to the α-carbon of the preceding residue in the sequence. The complete local frame is then established through orthogonalization. For each drug atom, a similar procedure is applied: The atom is taken as the origin, the *z* axis is oriented toward its nearest residue, and the *xz* plane is defined using the vector pointing to its nearest neighboring atom, with the orthogonalization step again used to construct the full local coordinate system. Subsequently, for each directed edge, we construct the following edge features:

$$e_{p\to d}^{\left(i,j\right)}=\left[{\left(p_{i}-q_{j}\right)}^{{\text{local}}_{j}},\;\phi \left(d_{\mathrm{ij}}\right),\;q_{\mathrm{ji}}^{\mathrm{rot}}\right]$$ (33)

$$e_{d\to p}^{\left(j,i\right)}=\left[{\left(q_{j}-p_{i}\right)}^{{\text{local}}_{i}},\;\phi \left(d_{\mathrm{ij}}\right),\;q_{\mathrm{ij}}^{\mathrm{rot}}\right]$$ (34)

where $p_{i}$ and $q_{j}$ denote the 3D coordinates of the residue and the drug atom, respectively. ${\left(p_{i}-q_{j}\right)}^{{\text{local}}_{j}}$ encodes, for a given drug atom j, the relative direction and distance of residue i with respect to it, and $\phi \left(d_{\mathrm{ij}}\right)$ is the Gaussian radial basis function encoding distance. $q_{\mathrm{ji}}^{\mathrm{rot}}$ represents the quaternion describing the rotation from the local coordinate frame defined by atom j to the local coordinate frame defined by residue i. $e_{d\to p}^{\left(j,i\right)}$ is defined similarly to the above.

Then, DTBind further re-encodes the individual residue and drug atom representations through graph convolutions on the heterogeneous graph to obtain the representation of the complex. Residue node features ${\left\{h_{i}^{\text{pocket}}\right\}}_{i=1}^{N_{\text{pocket}}}$ and drug node features ${\left\{h_{j}^{d}\right\}}_{j=1}^{N_{d}}$ are first projected into a hidden space for initialization:

$$h_{p,i}^{0}=\sigma \left(\mathrm{FC}\left(h_{i}^{\text{pocket}}\right)\right)\in R^{h_{6}}$$ (35)

$$h_{d,j}^{0}=\sigma \left(\mathrm{FC}\left(h_{j}^{d}\right)\right)\in R^{h_{6}}$$ (36)

$e_{p\to d}^{\left(i,j\right)}$ and $e_{d\to p}^{\left(j,i\right)}$ have also undergone a linear transformation to become $h_{6}$-dimensional.

We perform *T* layers of graph convolutional network, for a node i at layer t:

$$h_{p,i}^{\left(t+1\right)}=\sigma \left(W^{\left(p,t\right)}h_{p,i}^{\left(t\right)}+\sum_{j\in N_{d}\left(i\right)}\alpha_{\mathrm{ij}}^{\left(p\right)}\;\left[h_{d,j}^{\left(t\right)}\|e_{d\to p}^{\left(j,i\right)}\right]\right)$$ (37)

$$\alpha_{\mathrm{ji}}^{\left(p\right)}=\underset{j\in N_{d}\left(i\right)}{\text{softmax}}\left(\frac{{\left(W_{Q}^{\left(p,t\right)}h_{p,i}^{\left(t\right)}\right)}^{⊤}\left(W_{K}^{\left(p,t\right)}\left[h_{d,j}^{\left(t\right)}\|e_{d\to p}^{\left(j,i\right)}\right]\right)}{\sqrt{h_{6}}}\right)$$ (38)

where $W^{\left(p,t\right)},W_{Q}^{\left(p,t\right)}\in R^{h_{6}\times h_{6}}$, $W_{K}^{\left(p,t\right)}\in R^{h_{6}\times 2h_{6}}$ are learnable parameter matrices and $N_{d}\left(i\right)$ denote all drug atom neighbors of residue i.

The same applies to a drug atom i at layer t:

$$h_{d,j}^{\left(t+1\right)}=\sigma \left(W^{\left(d,t\right)}h_{d,i}^{\left(t\right)}+\sum_{j\in N_{p}\left(i\right)}\alpha_{\mathrm{ij}}^{\left(d\right)}\;\left[h_{p,j}^{\left(t\right)}\|e_{p\to d}^{\left(i,j\right)}\right]\right)$$ (39)

$$\alpha_{\mathrm{ij}}^{\left(d\right)}=\underset{j\in N_{p}\left(i\right)}{\text{softmax}}\left(\frac{{\left(W_{Q}^{\left(d,t\right)}h_{d,i}^{\left(t\right)}\right)}^{⊤}\left(W_{K}^{\left(d,t\right)}\left[h_{p,j}^{\left(t\right)}\|e_{p\to d}^{\left(i,j\right)}\right]\right)}{\sqrt{h_{6}}}\right)$$ (40)

where $W^{\left(d,t\right)},W_{Q}^{\left(d,t\right)}\in R^{h_{6}\times h_{6}}$, $W_{K}^{\left(d,t\right)}\in R^{h_{6}\times 2h_{6}}$ are learnable parameter matrices and $N_{p}\left(i\right)$ denote all residue neighbors of drug atom i.

After T layer graph convolution, we obtain final pocket–drug complex encoding $\begin{array}{c} \begin{array}{c} \begin{array}{c} H_{\text{complex}}^{\text{final}}={\left[h_{p,1}^{T},\cdots h_{N_{\text{pocket}}}^{T},\;h_{d,1}^{T},\cdots h_{N_{d}}^{T}\right]}^{T}\in R^{\left(N_{d}+N_{\text{pocket}}\right)\times h_{6}} \end{array} \end{array} \end{array}$. Subsequently, we use it to compute the feature of the protein within the complex, the drug within the complex, and the complex, respectively. Specifically, the protein feature within the complex is obtained by summing the features of all pocket residues in the complex, while the drug feature within the complex is obtained by summing the features of all drug atoms in the complex:

$$u_{p}=\sum_{i=1}^{N_{p}}h_{p,i}^{T},u_{d}=\sum_{j=1}^{N_{d}}h_{d,\;j}^{T}$$ (41)

The complex feature is formed by adaptively weighted aggregation of each residue in the pocket and each atom in the drug:

$$g=\sum_{i,j}\alpha_{\mathrm{ij}}\cdot W_{v}\left[h_{p,\;i}\|h_{d,\;j}\right]$$ (42)

$$\alpha_{\mathrm{ij}}=W_{\mathrm{att}}\left[h_{p,i}\|h_{d,j}\right]$$ (43)

where $W_{v}\in R^{h_{6}\times 2h_{6}}$ and $W_{\mathrm{att}}\in R^{1\times 2h_{6}}$ are learnable parameter matrices.

Finally, a classifier is used to obtain the predicted binding affinity:

$$\hat{y}=\text{AffPred}\left(g\|\;u_{d}\;\|\;\;u_{p}\right)$$ (44)

where AffPred(·) conforms the hidden dimension from $h_{6}\to 2h_{6}\to 1$.

The model was trained using hyperparameter configurations detailed in Table S10. Computational resource statistics are reported in Table S11, and the procedures for performance metric computation are described in Note S5.

## Data Availability

All data resources used in this study are freely available from the following databases: the PDBbind dataset (v2020) at http://www.pdbbind.org.cn/, the BioSnap dataset at https://github.com/kexinhuang12345/MolTrans/tree/master/dataset/BIOSNAP/full_data, and the RCSB Protein Data Bank at https://www.rcsb.org/. The source code for DTBind is openly available at https://github.com/liqy09/DTBind. Processed datasets required to reproduce and evaluate DTBind have been deposited in Zenodo (https://zenodo.org/records/17283638).

## Supplementary Materials

Supplementary 1 Notes S1 to S7 Tables S1 to S12

## References

[1] Bell TW, Hext NM. Supramolecular optical chemosensors for organic analytes. Chem Soc Rev. 2004;33:589–598. 15592624 10.1039/b207182g

[2] Ariga K, Ariga K, Kunitake T. *Supramolecular chemistry: Fundamentals and applications advanced textbook*. Heidelberg: Springer; 2006.

[3] Cragg PJ. *Supramolecular chemistry: From biological inspiration to biomedical applications*. Dordrecht, New York: Springer; 2010.

[4] Mareeswaran PM, Suresh P, Rajagopal S. *Photophysics of supramolecular architectures.* Southampton: Bentham Science Publishers; 2022.

[5] Lyu J, Wang S, Balius TE, Singh I, Levit A, Moroz YS, O’Meara MJ, Che T, Algaa E, Tolmachova K, et al. Ultra-large library docking for discovering new chemotypes. Nature. 2019;566:224–229. 30728502 10.1038/s41586-019-0917-9 PMC6383769

[6] Irwin JJ, Sterling T, Mysinger MM, Bolstad ES, Coleman RG. ZINC: A free tool to discover chemistry for biology. J Chem Inf Model. 2012;52(7):1757–1768. 22587354 10.1021/ci3001277 PMC3402020

[7] Gorgulla C, Boeszoermenyi A, Wang ZF, Fischer PD, Coote PW, Padmanabha das KM, Malets YS, Radchenko DS, Moroz YS, Scott DA, et al. An open-source drug discovery platform enables ultra-large virtual screens. Nature. 2020;580(7805):663–668. 32152607 10.1038/s41586-020-2117-z PMC8352709

[8] Binkowski TA, Joachimiak A. Protein functional surfaces: Global shape matching and local spatial alignments of ligand binding sites. BMC Struct Biol. 2008;8:45. 18954462 10.1186/1472-6807-8-45 PMC2626596

[9] Zhang Z, Quan L, Wang J, Peng L, Chen Q, Zhang B, Cao L, Jiang Y, Li G, Nie L, et al. LABind: Identifying protein binding ligand-aware sites via learning interactions between ligand and protein. Nat Commun. 2025;16(1):7712. 40830361 10.1038/s41467-025-62899-0 PMC12365077

[10] Liu Y, Yang X, Gan J, Chen S, Xiao Z-X, Cao Y. CB-Dock2: Improved protein-ligand blind docking by integrating cavity detection, docking and homologous template fitting. Nucleic Acids Res. 2022;50(W1):W159–W164. 35609983 10.1093/nar/gkac394 PMC9252749

[11] Utgés JS, Barton GJ. Comparative evaluation of methods for the prediction of protein-ligand binding sites. J Cheminform. 2024;16(1):126. 39529176 10.1186/s13321-024-00923-z PMC11552181

[12] DeMeester TR, Johnson LF. Evaluation of the Nissen antireflux procedure by esophageal manometry and twenty-four hour pH monitoring. Am J Surg. 1975;129(1):94–100. 2024 10.1016/0002-9610(75)90174-9

[13] Ahmed A, Mam B, Sowdhamini R. DEELIG: A deep learning approach to predict protein-ligand binding affinity. Bioinform Biol Insights. 2021;15: Article 11779322211030364. 34290496 10.1177/11779322211030364 PMC8274096

[14] Li Y, Su M, Liu Z, Li J, Liu J, Han L, Wang R. Assessing protein-ligand interaction scoring functions with the CASF-2013 benchmark. Nat Protoc. 2018;13(4):666–680. 29517771 10.1038/nprot.2017.114

[15] Zitnik M, Nguyen F, Wang B, Leskovec J, Goldenberg A, Hoffman MM. Machine learning for integrating data in biology and medicine: Principles, practice, and opportunities. Inf Fusion. 2019;50:71–91. 30467459 10.1016/j.inffus.2018.09.012 PMC6242341

[16] Schneider H-J. Binding mechanisms in supramolecular complexes. Angew Chem Int Ed Engl. 2009;48(22):3924–3977. 19415701 10.1002/anie.200802947

[17] Fink EA, Bardine C, Gahbauer S, Singh I, Detomasi TC, White K, Gu S, Wan X, Chen J, Ary B, et al. Large library docking for novel SARS-CoV-2 main protease non-covalent and covalent inhibitors. Protein Sci. 2023;32(8): Article e4712. 37354015 10.1002/pro.4712 PMC10364469

[18] Gossert AD, Jahnke W. NMR in drug discovery: A practical guide to identification and validation of ligands interacting with biological macromolecules. Prog Nucl Magn Reson Spectrosc. 2016;97:82–125. 27888841 10.1016/j.pnmrs.2016.09.001

[19] Orts J, Gossert AD. Structure determination of protein-ligand complexes by NMR in solution. Methods. 2018;138–139:3–25. 10.1016/j.ymeth.2018.01.019 29427713

[20] Schirò A, Carlon A, Parigi G, Murshudov G, Calderone V, Ravera E, Luchinat C. On the complementarity of X-ray and NMR data. J Struct Biol X. 2020;4: Article 100019. 32647823 10.1016/j.yjsbx.2020.100019 PMC7337059

[21] Hospital A, Goñi JR, Orozco M, Gelpí JL. Molecular dynamics simulations: Advances and applications. Adv Appl Bioinform Chem. 2015;8:37–47. 26604800 10.2147/AABC.S70333 PMC4655909

[22] Chen F, Gülbakan B, Weidmann S, Fagerer SR, Ibáñez AJ, Zenobi R. Applying mass spectrometry to study non-covalent biomolecule complexes. Mass Spectrom Rev. 2016;35(1):48–70. 25945814 10.1002/mas.21462

[23] Bagherian M, Sabeti E, Wang K, Sartor MA, Nikolovska-Coleska Z, Najarian K. Machine learning approaches and databases for prediction of drug-target interaction: A survey paper. Brief Bioinform. 2021;22(1):247–269. 31950972 10.1093/bib/bbz157 PMC7820849

[24] Wen M, Zhang Z, Niu S, Sha H, Yang R, Yun Y, Lu H. Deep-learning-based drug-target interaction prediction. J Proteome Res. 2017;16(4):1401–1409. 28264154 10.1021/acs.jproteome.6b00618

[25] Öztürk H, Özgür A, Ozkirimli E. DeepDTA: Deep drug-target binding affinity prediction. Bioinformatics. 2018;34(17):i821–i829. 30423097 10.1093/bioinformatics/bty593 PMC6129291

[26] Xie Z-R, Hwang M-J. Methods for predicting protein-ligand binding sites. Methods Mol Biol. 2015;1215:383–398. 25330972 10.1007/978-1-4939-1465-4_17

[27] Jeevan K, Palistha S, Tayara H, Chong KT. PUResNetV2.0: A deep learning model leveraging sparse representation for improved ligand binding site prediction. J Cheminform. 2024;16(1):66. 38849917 10.1186/s13321-024-00865-6 PMC11157904

[28] Bai P, Miljković F, John B, Lu H. Interpretable bilinear attention network with domain adaptation improves drug–target prediction. Nat Mach Intell. 2023;5:126–136.

[29] Nguyen T, Le H, Quinn TP, Nguyen T, Le TD, Venkatesh S. GraphDTA: Predicting drug-target binding affinity with graph neural networks. Bioinformatics. 2021;37(8):1140–1147. 33119053 10.1093/bioinformatics/btaa921

[30] Hadipour H, Li YY, Sun Y, Deng C, Lac L, Davis R, Cardona ST, Hu P. GraphBAN: An inductive graph-based approach for enhanced prediction of compound-protein interactions. Nat Commun. 2025;16:2541. 40102386 10.1038/s41467-025-57536-9 PMC11920434

[31] Jiang M, Li Z, Zhang S, Wang S, Wang X, Yuan Q, Wei Z. Drug–target affinity prediction using graph neural network and contact maps. RSC Adv. 2020;10(35):20701–20712. 35517730 10.1039/d0ra02297g PMC9054320

[32] Mylonas SK, Axenopoulos A, Daras P. DeepSurf: A surface-based deep learning approach for the prediction of ligand binding sites on proteins. Bioinformatics. 2021;37(12):1681–1690. 33471069 10.1093/bioinformatics/btab009

[33] Jiménez J, Doerr S, Martínez-Rosell G, Rose AS, De Fabritiis G. DeepSite: Protein-binding site predictor using 3D-convolutional neural networks. Bioinformatics. 2017;33(19):3036–3042. 28575181 10.1093/bioinformatics/btx350

[34] Renaud N, Geng C, Georgievska S, Ambrosetti F, Ridder L, Marzella DF, Reau MF, Bonvin AMJJ, Xue LC. DeepRank: A deep learning framework for data mining 3D protein-protein interfaces. Nat Commun. 2021;12(1):7068. 34862392 10.1038/s41467-021-27396-0 PMC8642403

[35] Karimi M, Wu D, Wang Z, Shen Y. DeepAffinity: Interpretable deep learning of compound-protein affinity through unified recurrent and convolutional neural networks. Bioinformatics. 2019;35(18):3329–3338. 30768156 10.1093/bioinformatics/btz111 PMC6748780

[36] Wang Z, Chen S, Zhang F, Akhmedov S, Weng J, Xu S. Prioritization of lipid metabolism targets for the diagnosis and treatment of cardiovascular diseases. Research. 2025;8:0618. 39975574 10.34133/research.0618 PMC11836198

[37] Peng L, Bai Z, Liu L, Yang L, Liu X, Chen M, Chen Z. DTI-MvSCA: An anti-over-smoothing multi-view framework with negative sample selection for predicting drug-target interactions. IEEE J Biomed Health Inform. 2025;29(1):711–723.

[38] Peng L, Liu X, Yang L, Liu L, Bai Z, Chen M, Lu X, Nie L. BINDTI: A bi-directional intention network for drug-target interaction identification based on attention mechanisms. IEEE J Biomed Health Inform. 2025;29(3):1602–1612. 38457318 10.1109/JBHI.2024.3375025

[39] Ru X, Xu L, Han W, Zou Q. In silico methods for drug-target interaction prediction. Cell Rep Methods. 2025;5(10): Article 101184. 40997795 10.1016/j.crmeth.2025.101184 PMC12570315

[40] Shang Y, Gao L, Zou Q, Yu L. Prediction of drug-target interactions based on multi-layer network representation learning. Neurocomputing. 2021;434:80–89.

[41] Ding Y, Tang J, Guo F, Zou Q. Identification of drug-target interactions via multiple kernel-based triple collaborative matrix factorization. Brief Bioinform. 2022;23(2): Article bbab582. 35134117 10.1093/bib/bbab582

[42] Huang K, Xiao C, Glass LM, Sun J. MolTrans: Molecular interaction transformer for drug–target interaction prediction. Bioinformatics. 2021;37(6):830–836. 33070179 10.1093/bioinformatics/btaa880 PMC8098026

[43] Luo Y, Liu Y, Peng J. Calibrated geometric deep learning improves kinase–drug binding predictions. Nat Mach Intell. 2023;5(12):1390–1401. 38962391 10.1038/s42256-023-00751-0 PMC11221792

[44] Leskovec J, Sosič R. SNAP: A general-purpose network analysis and graph-mining library. ACM Trans Intell Syst Technol. 2017;8:1–20. 10.1145/2898361 PMC5361061 28344853

[45] Lee I, Keum J, Nam H. DeepConv-DTI: Prediction of drug-target interactions via deep learning with convolution on protein sequences. PLOS Comput Biol. 2019;15(6): Article e1007129. 31199797 10.1371/journal.pcbi.1007129 PMC6594651

[46] Cortes C, Vapnik V. Support-vector networks. Mach Learn. 1995;20:273–297.

[47] Liu Z, Li Y, Han L, Li J, Liu J, Zhao Z, et al. PDB-wide collection of binding data: Current status of the PDBbind database. Bioinformatics. 2015;31(3):405–412. 25301850 10.1093/bioinformatics/btu626

[48] Krivák R, Hoksza D. P2Rank: Machine learning based tool for rapid and accurate prediction of ligand binding sites from protein structure. J Cheminform. 2018;10(1):39. 30109435 10.1186/s13321-018-0285-8 PMC6091426

[49] Wang J, Liu Y, Tian B. Protein-small molecule binding site prediction based on a pre-trained protein language model with contrastive learning. J Cheminform. 2024;16:125. 39506806 10.1186/s13321-024-00920-2 PMC11542454

[50] Lu Z, Song G, Zhu H, Lei C, Sun X, Wang K, Qin L, Chen Y, Tang J, Li M. DTIAM: A unified framework for predicting drug-target interactions, binding affinities and drug mechanisms. Nat Commun. 2025;16(1):2548. 40089473 10.1038/s41467-025-57828-0 PMC11910601

[51] Li S, Wan F, Shu H, Jiang T, Zhao D, Zeng J. MONN: A multi-objective neural network for predicting compound-protein interactions and affinities. Cell Syst. 2020;10(4):308–322.e11.

[52] Su M, Yang Q, Du Y, Feng G, Liu Z, Li Y, Wang R. Comparative assessment of scoring functions: The CASF-2016 update. J Chem Inf Model. 2019;59(2):895–913. 30481020 10.1021/acs.jcim.8b00545

[53] Elnaggar A, Heinzinger M, Dallago C, Rehawi G, Wang Y, Jones L, Gibbs T, Feher T, Angerer C, Steinegger M, et al. ProtTrans: Toward understanding the language of life through self-supervised learning. IEEE Trans Pattern Anal Mach Intell. 2022;44(10):7112–7127. 34232869 10.1109/TPAMI.2021.3095381

[54] Lin Z, Akin H, Rao R, Hie B, Zhu Z, Lu W, Smetanin N, Verkuil R, Kabeli O, Shmueli Y, et al. Evolutionary-scale prediction of atomic-level protein structure with a language model. Science. 2023;379(6637):1123–1130. 36927031 10.1126/science.ade2574

[55] Selvaraju RR, Cogswell M, Das A, Vedantam R, Parikh D, Batra D. Grad-CAM: Visual explanations from deep networks via gradient-based localization. In: *2017 IEEE International Conference on Computer Vision (ICCV)*. Venice: IEEE; 2017. p. 618–626.

[56] Engh RA, Girod A, Kinzel V, Huber R, Bossemeyer D. Crystal structures of catalytic subunit of cAMP-dependent protein kinase in complex with isoquinolinesulfonyl protein kinase inhibitors H7, H8, and H89. Structural implications for selectivity. J Biol Chem. 1996;271(42):26157–26164. 8824261 10.1074/jbc.271.42.26157

[57] Armstrong N, Gouaux E. Mechanisms for activation and antagonism of an AMPA-sensitive glutamate receptor: Crystal structures of the GluR2 ligand binding core. Neuron. 2000;28(1):165–181. 11086992 10.1016/s0896-6273(00)00094-5

[58] Pantoom S, Vetter IR, Prinz H, Suginta W. Potent family-18 chitinase inhibitors: X-ray structures, affinities, and binding mechanisms. J Biol Chem. 2011;286(27):24312–24323. 21531720 10.1074/jbc.M110.183376 PMC3129211

[59] Tao Z-F, Hasvold LA, Leverson JD, Han EK, Guan R, Johnson EF, et al. Discovery of 3H-benzo[4,5]thieno[3,2-d]pyrimidin-4-ones as potent, highly selective, and orally bioavailable inhibitors of the human protooncogene proviral insertion site in moloney murine leukemia virus (PIM) kinases. J Med Chem. 2009;52(21):6621–6636. 19842661 10.1021/jm900943h

[60] Salentin S, Schreiber S, Haupt VJ, Adasme MF, Schroeder M. PLIP: Fully automated protein–ligand interaction profiler. Nucleic Acids Res. 2015;43(W1):W443–W447. 25873628 10.1093/nar/gkv315 PMC4489249

[61] Liu T, Lin Y, Wen X, Jorissen RN, Gilson MK. BindingDB: A web-accessible database of experimentally determined protein-ligand binding affinities. Nucleic Acids Res. 2007;35:D198–D201. 17145705 10.1093/nar/gkl999 PMC1751547

[62] UniProt Consortium. UniProt: A worldwide hub of protein knowledge. Nucleic Acids Res. 2019;47(D1):D506–D515. 30395287 10.1093/nar/gky1049 PMC6323992

[63] Varadi M, Anyango S, Deshpande M, Nair S, Natassia C, Yordanova G, Yuan D, Stroe O, Wood G, Laydon A. AlphaFold protein structure database: Massively expanding the structural coverage of protein-sequence space with high-accuracy models. Nucleic Acids Res. 2022;50(D1):D439–D444. 34791371 10.1093/nar/gkab1061 PMC8728224

[64] Pronk S, Páll S, Schulz R, Larsson P, Bjelkmar P, Apostolov R, Shirts MR, Smith JC, Kasson PM, van der Spoel D, et al. GROMACS 4.5: A high-throughput and highly parallel open source molecular simulation toolkit. Bioinformatics. 2013;29(7):845–854. 23407358 10.1093/bioinformatics/btt055 PMC3605599

[65] Jumper J, Evans R, Pritzel A, Green T, Figurnov M, Ronneberger O, Tunyasuvunakool K, Bates R, Zidek A, Potapenko A, et al. Highly accurate protein structure prediction with AlphaFold. Nature. 2021;596(7873):583–589. 34265844 10.1038/s41586-021-03819-2 PMC8371605

[66] Mou M, Zhang Z, Pan Z, Zhu F. Deep learning for predicting biomolecular binding sites of proteins. Research. 2025;8:0615. 39995900 10.34133/research.0615 PMC11848751

[67] Guan M, Han J, Zhang S, Zheng H, Liu J. SpatConv enables the accurate prediction of protein binding sites by a pretrained protein language model and an interpretable bio-spatial convolution. Research. 2025;8:0773. 40636133 10.34133/research.0773 PMC12237623

[68] Zhang S, Han J, Liu J. Protein–protein and protein–nucleic acid binding site prediction via interpretable hierarchical geometric deep learning. GigaScience. 2024;13: Article giae080. 39484977 10.1093/gigascience/giae080 PMC11528319

[69] Han J, Zhang S, Guan M, Li Q, Gao X, Liu J. GeoNet enables the accurate prediction of protein-ligand binding sites through interpretable geometric deep learning. Structure. 2024;32(12):2435–2448.e5. 39488202 10.1016/j.str.2024.10.011

[70] Xu Z, Zhu Y, Han J, Liu J. SpatPPI: A geometric deep learning model for predicting protein–protein interactions involving intrinsically disordered regions. Genome Biol. 2025;26:339. 41053908 10.1186/s13059-025-03820-2 PMC12498438

[71] Berman HM, Westbrook J, Feng Z, Gilliland G, Bhat TN, Weissig H, et al. The Protein Data Bank. Nucleic Acids Res. 2000;28(1):235–242. 10592235 10.1093/nar/28.1.235 PMC102472

[72] Kim S, Thiessen PA, Bolton EE, Chen J, Fu G, Gindulyte A, et al. PubChem substance and compound databases. Nucleic Acids Res. 2016;44(D1):D1202–D1213. 26400175 10.1093/nar/gkv951 PMC4702940

[73] Chai Discovery Team, Boitreaud J, Dent J, McPartlon M, Meier J, Reis V, Rogozhonikov A, Wu K. Chai-1: Decoding the molecular interactions of life. bioRxiv. 2024. 10.1101/2024.10.10.615955

[74] Landrum G, et al. RDKit: Open-source cheminformatics. 2006. https://github.com/rdkit/rdkit

