# Supplementary material for: DTBind: A Mechanism-Driven Deep Learning Framework for Accurate Prediction of Drug–Target Molecular Recognition
Source: Research (Wash D C). 2025 Dec 2;8:1022. doi: 10.34133/research.1022 (PMC12669800; doi:10.34133/research.1022)
Supplement: Supplementary 1 — Notes S1 to S7 Tables S1 to S12 [file research.1022.f1.pdf]

Supplementary Information

DTBind: a mechanism-driven deep learning framework for accurate prediction of drug-target molecular recognition

Qiuyu Li[1,†], Zeyu Xu[1,†], Yanhao Zhu[1,†], Wanyun Zhou[2], Mingming Guan[1], Shiqing Zhao[1], Mengyuan Liu[1], Bin Liu[3,*], Juntao Liu[1,*]

[1]School of Mathematics and Statistics, Shandong University, Weihai, 264209, China

[2]The Hong Kong University of Science and Technology (Guangzhou), Guangzhou, 511453, P.R.China

[3]Department of Neurosurgery, Shandong Provincial Hospital Affiliated to Shandong First Medical University, Jinan 250021, China

[†]These authors contributed equally to this study

[*]To whom correspondence should be addressed

Email addresses:

JL: juntaosdu@126.com

BL: binliu@email.sdfmu.edu.cn

Supplementary Contents

Supplementary Tables

Supplementary Notes

References

Supplementary Tables

Table S1. Performance (AUROC, AUPRC, and F1-score) of DTBind and other compared methods on the Bind Occurrence Prediction.

**Method**	AUROC	AUPRC	F1-score
DeepConvDTI	0.886 ± 0.006	0.890 ± 0.006	0.792 ± 0.020
DrugBAN	0.903 ± 0.005	0.902 ± 0.004	0.843 ± 0.008
MolTrans	0.895 ± 0.004	0.897 ± 0.005	0.816 ± 0.025
SVM	0.862 ± 0.007	0.864 ± 0.004	0.764 ± 0.026
DTBind	**0.935 ± 0.001**	**0.940 ± 0.006**	**0.872 ± 0.003**

Table S2. Performance (MCC, Accuracy, and Sensitivity) of DTBind and other compared methods on the Bind Occurrence Prediction.

**Method**	MCC	Accuracy	Sensitivity
DeepConvDTI	0.601 ± 0.008	0.805 ± 0.009	0.760 ± 0.029
DrugBAN	0.672 ± 0.018	0.834 ± 0.008	0.820 ± 0.021
MolTrans	0.625 ± 0.033	0.825 ± 0.010	0.818 ± 0.031
SVM	0.560 ± 0.017	0.777 ± 0.011	0.711 ± 0.042
DTBind	**0.740 ± 0.005**	**0.870 ± 0.002**	**0.841 ± 0.013**

Table S3. Performance of DTBind and other compared methods on the Bind Site Prediction.

**Method**	F1-score	MCC	ACC	Recall	Precision	AUROC
DeepSurf	0.355	0.403	0.939	0.777	0.230	0.860
P2Rank	0.173	0.259	0.823	**0.897**	0.096	0.859
RUResNet	0.417	0.468	0.951	0.848	0.276	0.901
CLAPE-SMB	0.225	0.208	0.967	0.229	0.222	0.606
DTBind	**0.589 ± 0.001**	**0.581 ± 0.001**	**0.982 ± 0.001**	0.635 ± 0.012	**0.550 ± 0.010**	**0.948 ± 0.005**

Table S4. Performance of DTBind and other compared methods on the Bind Affinity Prediction.

**Method**	RMSE	Pearson	Spearman	MAE
GraphDTA	1.780 ± 0.008	0.549 ± 0.017	0.640 ± 0.018	1.420 ± 0.027
DeepDTA	2.681 ± 0.342	0.746 ± 0.012	0.723 ± 0.015	2.155 ± 0.028
DTIAM	1.288 ± 0.008	0.791 ± 0.004	0.776 ± 0.006	1.009 ± 0.007
MONN	1.842 ± 0.084	0.391 ± 0.006	0.435 ± 0.012	1.425 ± 0.042
KDBNet	6.767 ± 0.030	0.486 ± 0.062	0.500 ± 0.061	6.415 ± 0.030
DTBind	**1.235 ± 0.005**	**0.806 ± 0.004**	**0.795 ± 0.005**	**0.981 ± 0.013**

Table S5. RMSE of DTBind and Other comparison methods on the Affinity Prediction test set is divided into different protein families.

**Method**	Kinase	Ion channel	Nuclear receptor	Serine proteases	Immuoglobulin	Other
GraphDTA	1.472	1.474	1.455	1.475	1.464	1.452
DeepDTA	1.934	2.091	2.086	3.294	2.385	2.667
DTIAM	**1.045**	2.030	1.336	1.421	1.567	**1.288**
MONN	1.597	2.980	2.047	1.443	2.716	2.021
KDBNet	7.232	8.086	8.179	7.240	7.216	6.319
DTBind	1.047	**1.150**	**1.331**	**0.925**	**0.851**	1.297

Table S6. Pearson's correlation coefficient of DTBind and Other comparison methods on the Affinity Prediction test set is divided into different protein families.

**Method**	Kinase	Ion channel	Nuclear receptor	Serine proteases	Immuoglobulin	Other
GraphDTA	0.622	0.618	0.633	0.607	0.622	0.622
DeepDTA	0.653	0.722	0.622	0.702	0.847	0.720
DTIAM	**0.739**	0.734	**0.745**	0.894	0.812	**0.782**
MONN	0.445	0.563	0.527	0.825	-0.117	0.441
KDBNet	0.245	**0.765**	0.428	0.640	0.437	0.401
DTBind	0.730	0.672	0.703	**0.958**	**0.913**	0.775

Table S7. Performance Metrics of Ablation Studies with Different Settings on the Bind Occurrence Prediction.

Ablation	F1	MCC	AUROC	AUPRC	Accuracy	Sensitivity	Specificity
w/o edge	0.851	0.691	0.917	0.923	0.845	0.850	0.822
w/o surface	0.858	0.712	0.921	0.923	0.856	0.854	0.858
w/o interaction	0.841	0.687	0.915	0.924	0.843	0.817	0.870
w/o gate	0.845	0.683	0.915	0.922	0.841	0.851	0.832
ESM2 feat	0.853	0.704	0.920	0.929	0.852	0.842	0.862
original	0.863	0.721	0.926	0.932	0.860	0.864	0.857

Note: ESM2 feat means replacing ProtT5 pretrained features with ESM2 pretrained features.

Table S8. Performance Metrics of Ablation Studies with Different Settings on the Binding Site Prediction.

Ablation	F1	MCC	Recall	Precision	Accuracy	AUROC	AUPRC
w/o edge	0.564	0.555	0.589	0.541	0.981	0.960	0.568
w/o surface	0.565	0.555	0.576	0.554	0.981	0.957	0.564
w/o interaction	0.566	0.558	0.629	0.514	0.980	0.960	0.568
w/o gate	0.575	0.567	0.634	0.525	0.980	0.958	0.578
ESM2 feat	0.584	0.581	0.704	0.500	0.976	0.957	0.593
original	0.589	0.581	0.641	0.545	0.981	0.958	0.578

Note: ESM2 feat means replacing ProtT5 pretrained features with ESM2 pretrained features.

Table S9. Performance Metrics of Ablation Studies with Different Settings on the Binding Affinity Prediction.

Ablation	RMSE	Pearson	Spearman	MAE
ESM2 feat	1.335	0.766	0.760	1.040
w/o surface	1.407	0.738	0.738	1.146
w/o HeteroGraph	1.363	0.752	0.749	1.085
w/o edge	1.350	0.762	0.741	1.078

original	1.223	0.810	0.795	0.968

Note: ESM2 feat means replacing ProtT5 pretrained features with ESM2 pretrained features.

Table S10. The Optimal Hyperparameter Settings.

Hyperparameters (binding occurrence)	number	Hyperparameters (binding site)	number	Hyperparameters (binding affinity)	number
h_1	128	h_1	128	h_1	128
h_2	32	h_2	32	h_2	32
h_3	128	h_3	128	h_3	128
h_4	128	h_5	128	h_6	128
Protein GCN layer	3	Protein GCN layer	3	Pocket GCN layer	3
Drug GCN layer	3	Drug GCN layer	3	Drug GCN layer	3
MCA head	4	MCA head	4	Hetero GCN layer	3
MSA head	4	MSA head	4	Batch size	32
Batch size	32	Batch size	16	Learning rate	0.0005
Learning rate	0.0005	Learning rate	0.0005	dropout rate	0.1
dropout rate	0.1	dropout rate	0.1	epoch	80
epoch	60	epoch	70		

Note: Because the protein feature encoder and drug feature encoder of the three prediction tasks are consistent, the three tasks share three hyperparameters h_1, h_2 and h_3, representing the hidden layer dimensions of the protein geometric encoder, protein surface feature encoder and drug feature encoder, respectively.

Table S11. Model computing resource statistics.

**Metrics**	Binding Occurrence	Binding Site	Binding Affinity
Total GPU Memory	23.7 GB	47.54 GB	39.49 GB
Total Parameters	1,100,033	895,458	2,312,938
Model Size	4.20 MB	3.42 MB	8.82 MB
Average Training Time per Epoch	~251.01s	~647.57s	~103.61s
Average Inference Time per Batch	~53.79ms	~54.74ms	~0.35s

Note: The above data is the result of statistics conducted on GPU type NVIDIA GeForce RTX 3090 (CUDA version 11.6).

Supplementary Notes

Supplementary Note 1 | Residue-level Gradient-weighted Class Activation Mapping

To assess the interpretability of DTBind's hierarchical protein representations, we applied a residue-level Gradient-weighted Class Activation Mapping (Grad-CAM) strategy[1] across three successive stages of the protein encoder: (i) the ProtT5 embedding layer, where residues are initialized with pretrained language-model features; (ii) the geometric graph encoder, which integrates local structural dependencies through edge-based message passing; and (iii) the fusion layer, where graph embeddings are combined with surface descriptors to form the final representation. These stages reflect, respectively, hysicochemical properties, functional characteristics, structural complementarity, and surface accessibility.

For a target layer with activation vectors $h_i \in R^C$, where N is the number of residues and C the number of feature channels, the model's residue-level prediction is denoted as y_j. To generate Grad-CAM maps, we define the objective $S = \sum_{j=1}^{N} y_j$ and compute gradients gic=$g_i^c = \frac{\partial S}{\partial h_i^c}$. Channel-wise importance weights are obtained via global average pooling,

$$\alpha_c = \frac{1}{N} \sum_{i=1}^{N} g_i^c,$$

which quantify the overall relevance of channel ccc. Residue-level importance scores are then given by

$$\mathrm{CAM_i} = \mathrm{ReLU}\left(\sum_{c=1}^{C} \alpha_c \; h_i^c\right), i = 1, \ldots, N,$$

where the ReLU operator retains positive contributions supporting binding predictions. The resulting CAM values are rescaled using min–max normalization to facilitate comparability across proteins of varying lengths.

This stepwise procedure revealed a progressive refinement of residue-level localization. At the embedding-only layer, CAM activations were diffuse and often assigned high importance to non-binding residues, reflecting broad biochemical relevance but limited specificity. At the graph-encoded layer, activations became more concentrated, highlighting residues consistent with local structural complementarity. At the final surface feature fusion layer, activations focused almost exclusively on true binding residues, demonstrating that surface accessibility

and pocket morphology provide essential cues for ligand recognition. Representative examples of these residue-level activation profiles are provided in the main text (Fig. 6).

To quantify how strongly true binding residues are concentrated among residues with the highest Grad-CAM activations, we defined a binding enrichment score that compares the frequency of annotated binding residues in the top-ranked subset to their frequency in the entire protein sequence background. Let:

N_{all}: total number of residues in the dataset;

N_{bind}: number of experimentally annotated binding residues;

N_{top}: number of residues within the top α% of Grad-CAM activations (here $\alpha = 5$);

$N_{bind,top}$: number of binding residues within this top α% subset.

The binding ratio and fold enrichment are computed as:

$$\text{Binding ratio}(\text{Top} - \alpha\%) = \frac{N_{bind,top}}{N_{top}}$$

$$\text{Enrichment}(\text{Top} - \alpha\%) = \frac{\text{Binding ratio}(\text{Top} - \alpha\%)}{N_{bind}/N_{all}}$$

In this formulation, the numerator represents the proportion of true binding residues among the highest-activation positions, while the denominator reflects the global background binding ratio. The resulting fold enrichment indicates how many times more frequently true binding residues occur among top-activated residues than expected by random distribution.

In our dataset, the background binding residue ratio (N_{bind}/N_{all}) is approximately 2.8%. The enrichment values were calculated by first computing the enrichment for each individual protein sample separately, and then averaging these per-sample enrichment values across the entire dataset. This sample-wise averaging approach ensures that each protein contributes equally to the final enrichment metric, regardless of its size or the number of binding residues it contains.

Therefore, an enrichment of 10× means that binding residues are ten times more prevalent within the top 5% of Grad-CAM activations than expected by chance, signifying a strong correspondence between model attention and true binding sites.

Supplementary Note 2 | Docking and MD Validation of DTBind Binding-Site Predictions in Proteins without Experimental Structures

Representative drug–target pairs with confirmed binding interactions were randomly selected from the BindingDB database[2]. For each protein, the corresponding UniProt[3] identifier was retrieved based on its amino acid sequence, and the predicted three-dimensional structure was obtained from the AlphaFold Protein Structure Database[4]. Ligand structures were collected from PubChem[5] by querying with the SMILES strings of the compounds, and the corresponding SDF files were downloaded. Using these AlphaFold-predicted protein structures together with the ligand SDF files, potential binding sites were first predicted with DTBind.

To evaluate the reliability of the DTBind-predicted binding sites, molecular docking was performed with AutoDock 4.2.6[6], followed by molecular dynamics (MD) simulations using GROMACS 2024.3[7]. The docked complexes were refined and equilibrated through MD simulations, and the final stable binding modes were analyzed to determine whether the DTBind-predicted residues were located within the docking pocket and in direct contact with the ligand.

Docking input preparation and parameter configuration were performed with AutoDock Tools (ADT) 1.5.7. Proteins were preprocessed by removing all crystallographic water molecules, co-crystallized ligands, and irrelevant ions. Polar hydrogens were added and Kollman united atom charges were assigned in ADT. Ligand molecules were parameterized by assigning Gasteiger charges in ADT. The docking grid box was defined to cover the entire protein. Docking was conducted using the Lamarckian Genetic Algorithm (LGA)[6] with the following parameters:

Table S12 docking parameters

Parameters	Value
Number of GA runs	50
Population size	150
Maximum number of generations	27,000
Maximum number of evals	2,500,000
GA crossover mode	twopt

For each docking run, 10 lowest-energy conformations were generated, and the protein–ligand complex whose binding site was closest to the DTBind-predicted pocket was selected for MD simulations.

Molecular dynamics simulations were performed with GROMACS 2024.3[7]. Proteins were parameterized with the AMBER ff14SB force field, and ligand parameters were generated using the GAFF2 force field. Partial charges were assigned with the AM1-BCC method, and topology files were generated using sobtop. Each complex was solvated in a cubic box of TIP3P water molecules, ensuring a minimum distance of 0.8 nm from the protein surface to the box boundary. Na^+ and Cl^- ions were added to neutralize the system and adjust the ionic strength to 0.15 M.

The system was first minimized using the steepest descent method until the maximum force fell below 1000 kJ/mol/nm. This was followed by two equilibration steps: (i) NVT equilibration for 100 ps at 300 K using the V-rescale thermostat, and (ii) NPT equilibration for 100 ps at 300 K and 1 bar using the Parrinello–Rahman barostat. During equilibration, positional restraints were applied to all heavy atoms of the protein–ligand complex. Production simulations were performed for 50 ns under NPT conditions, with a 2 fs integration step. All covalent bond lengths were constrained using the LINCS algorithm, while thermostat and barostat settings were the same as in the NPT equilibration phase.

After ~30 ns, the complexes reached equilibrium, as indicated by the convergence of backbone RMSD to a stable plateau of ~2.5 Å, which was maintained throughout the remainder of the simulation. Analysis of the equilibrated structures confirmed that the DTBind-predicted binding residues were consistently localized within the docking pockets and maintained close contacts with the ligands, supporting the reliability of the DTBind predictions.

Supplementary Note 3 | Detailed Construction of Local Residue Coordinate Frames

This note provides the detailed mathematical procedure for constructing the local, intrinsic orthonormal coordinate frame for each amino acid residue, as used in the protein graph representation.

For each residue i, we construct an intrinsic, residue-centred orthonormal frame $O_i = [x_i, y_i, z_i] \in R^{3\times3}$ with origin at the α carbon atom coordinate $c_i^\alpha \in R^3$ (if c_i^α is missing we use the residue centroid) and carbonyl-carbon atom coordinate $c_i^C \in R^3$. The local z-axis is taken along the direction from the α carbon toward the carbonyl carbon,

$$z_i = \frac{c_i^C - c_i^\alpha}{\left\|c_i^C - c_i^\alpha\right\|_2}.$$

To define the xz-plane we use the forward backbone direction,

$$p_i = c_{i+1}^\alpha - c_i^\alpha,$$

with the obvious fallback $p_i = c_i^\alpha - c_{i-1}^\alpha$ at chain termini. The local y-axis is the (normalized) cross product of z_i and p_i,

$$\mathrm{y}_i = \frac{\mathrm{z}_i \times \mathrm{p}_i}{\|\mathrm{z}_i \times \mathrm{p}_i\|_2},$$

and the local x-axis is obtained by

$$\mathrm{x}_i = \frac{\mathrm{y}_i \times \mathrm{z}_i}{\|\mathrm{y}_i \times \mathrm{z}_i\|_2}.$$

This construction yields an intrinsic, residue-centered coordinate system that (i) reflects local backbone orientation and side-chain directionality and (ii) enforces invariance to global rigid-body motions.

To ensure numerical stability in constructing local residue coordinate frames and their quaternion representations, we apply the following strategies:

Robust Handling of Degenerate Vector Configurations: Our framework proactively detects and mitigates numerical instability arising from near-zero or near-collinear vectors. This is achieved through threshold-based checks on vector magnitudes and cross-product norms. In these degenerate cases, the algorithm employs predefined default directions to initialize a stable orthonormal basis, which is then rigorously enforced through successive cross-product operations.

Normalization of Rotation Representations: In constructing local coordinate frames, all vectors used for generating rotation matrices are normalized to unit length. The resulting rotation matrices are constrained to be orthonormal, and their corresponding quaternions are normalized to unit norm, ensuring numerical stability and geometric validity across all transformations.

Supplementary Note 4 | Multi-head Cross Attention and Self-Attention

Multi-head cross-attention (MCA) extends the standard scaled dot-product attention mechanism by enabling interactions between two distinct feature spaces (e.g., protein and drug embeddings). In each head, the attention operation is defined as:

$$\text{Attention}(Q, K, V) = \text{softmax}\left(\frac{QK^{\top}}{\sqrt{d_k}}\right)V,$$

where Q, K, and V denote the query, key, and value matrices, respectively, d is the hidden dimension of the fused representation space used in the fusion modules, and $d_k = d/k$ is the dimensionality of each head.

To capture complementary interaction patterns, multi-head attention projects the input matrices into k distinct subspaces, performs attention in parallel, and then aggregates the results. For the i-th head:

$$\text{head}_i = \text{Attention}(Q, K, V),$$

The outputs from all heads are concatenated and mapped back to the original feature dimension:

$$\text{MHA}(Q, K, V) = [\text{head}_1 || \cdots || \text{head}_k] W_O,$$

with $W_O \in R^{kd_v \times d}$ being a learnable output projection, $d_v = d/k$ is the per-head dimension of the value matrix V.

In the case of multi-head self-attention (MSA), the query, key, and value matrices are derived from the same input representation, allowing the model to capture contextual dependencies within a single modality. In contrast, multi-head cross-attention (MCA) computes attention across two different modalities (e.g., drug and protein), thereby facilitating fine-grained interaction modeling between heterogeneous representations.

Supplementary Note 5 | Evaluation of multi-level prediction

To comprehensively assess the performance of our multi-level model, we evaluate each task using established metrics tailored to the nature of the prediction targets. Specifically, we consider three tasks: (i) drug–target binding affinity prediction, (ii) binding occurrence prediction (whether a drug binds a target), and (iii) residue-level binding site prediction.

Binding affinity prediction. For the regression task of predicting drug–target binding affinity, we employ the following metrics: Root Mean Squared Error (RMSE), Mean Absolute Error (MAE), Pearson correlation coefficient (r)}, and Spearman's rank correlation coefficient (ρ)}:

$$\text{RMSE} = \sqrt{\frac{1}{N}\sum_{i=1}^{N}(y_i - \hat{y}_i)^2},$$

$$\mathrm{MAE} = \frac{1}{N}\sum_{i=1}^{N} |y_i - \hat{y}_i|,$$

$$r = \frac{\sum_{i=1}^{N} (y_i - \bar{y})(\hat{y}_i - \bar{\hat{y}})}{\sqrt{\sum_{i=1}^{N} (y_i - \bar{y})^2}\sqrt{\sum_{i=1}^{N} (\hat{y}_i - \bar{\hat{y}})^2}},$$

$$\rho = 1 - \frac{6\sum_{i=1}^{N} d_i^2}{N(N^2 - 1)},$$

where y_i and $\hat{y}_i$ denote the ground-truth and predicted affinities for sample i, N is the number of samples, $\bar{y}$ and $\bar{\hat{y}}$ are the mean values of true and predicted affinities, and d_i is the rank difference of the i-th sample.

Binding occurence prediction. For predicting whether a drug binds a protein (binary classification), we employ F1-score, Matthews Correlation Coefficient (MCC), Area Under the Precision–Recall Curve (AUPRC), Area Under the Receiver Operating Characteristic Curve (AUROC), Accuracy, Sensitivity (Recall), and Specificity. Given true positives (TP), true negatives (TN), false positives (FP), and false negatives (FN):

$$\text{Precision}=\frac{\text{TP}}{\text{TP+FP}},$$

$$\text{Recall}=\frac{\text{TP}}{\text{TP+FN}},$$

$$\text{F1-score}=2\cdot\frac{\text{Precision}\cdot\text{Recall}}{\text{Precision+Recall}},$$

$$\text{MCC}=\frac{\text{TP}\cdot\text{TN-FP}\cdot\text{FN}}{\sqrt{(\text{TP+FP})(\text{TP+FN})(\text{TN+FP})(\text{TN+FN})}},$$

$$\text{Accuracy}=\frac{\text{TP+TN}}{\text{TP+TN+FP+FN}},$$

$$\text{Sensitivity=Recall},$$

$$\text{Specificity}=\frac{\text{TN}}{\text{TN+FP}}.$$

AUPRC and AUROC are computed based on varying classification thresholds to summarize performance across the precision–recall and ROC curves, respectively.

Binding site prediction. Binding site prediction is also formulated as a binary classification task, but at the residue level rather than the pair level. For each drug–target pair, the model outputs a probability for every residue being a binding site. These predictions are then compared with residue-level ground-truth labels, and the same classification metrics as

above (F1, MCC, AUPRC, AUROC, Accuracy, Sensitivity, Specificity) are applied. To compute these metrics, predictions across all residues are aggregated, treating each residue as an individual sample.

Decision Threshold Optimization. For both classification tasks, the decision threshold was determined based on the optimal Matthews Correlation Coefficient (MCC). pecifically, threshold values ranging from 0.02 to 0.98 (with a step size of 0.02) were systematically evaluated on the validation set, and the threshold achieving the highest MCC was selected for final metric computation. All threshold-dependent classification metrics, including F1-score, precision, recall, specificity, accuracy, and the MCC itself, were computed using predictions generated at this optimized threshold.

Supplementary Note 6 | Surface Feature Extraction

To extract residue-level surface descriptors, we adopted a methodology inspired by GeoBind[8] and dMaSIF[9], focusing on local geometric and chemical features derived from the molecular surface. The surface feature extraction pipeline follows these key steps:

1. Molecular Surface Generation

The solvent-excluded molecular surface was generated using the MSMS program (Michel Sanner's Molecular Surface, version 2.6.1). Protein structures were first protonated using Reduce (Word et al., 1999), and atomic radii were assigned according to standard element types (C, N, O, S, P, H). The solvent-excluded surface was triangulated with a probe radius of 1.5 Å and a vertex density of 3, resulting in a mesh of vertices and faces. The meshes were resampled at a resolution of 1.2 Å using PyMESH to ensure uniform vertex spacing and to remove isolated components.

2. Geometric Surface Feature Computation

For each surface vertex, geometric properties such as mean curvature (H), Gaussian curvature (K), shape index (S), and curvedness (C) were computed using discrete differential geometry operators from trimesh. The shape index and curvedness were calculated as:

$$S = \frac{2}{\pi}\arctan\left(\frac{H}{K}\right),\ C = \sqrt{H^2 + K^2}$$

3. Final Residue-Level Feature Representation

Each surface vertex was mapped to its nearest residue. The geometric features (H, K, S, C)

of all the surface vertices mapped to it were aggregated (averaged) to form a 4-dimensional geometric feature vector only for residues located near the protein surface. Simultaneously, each residue was represented by a 6-dimensional physicochemical feature vector S_{chem} obtained by summing the one-hot encodings of the atom types (C, H, O, N, S, Other) of all atoms comprising the residue, thereby capturing the overall atomic composition at the residue level. The final 10-dimensional residue-level surface feature vector is obtained by concatenating the 4D geometric and 6D chemical descriptors:

$$S = [S_{chem}, H, K, S, C] \in R^{10}$$

For non-surface residues, the geometric features are set to zero (chemical features were extracted normally), as they are not exposed to the surface.

Note 7. Handling Class Imbalance with Focal Loss

The binding-site dataset exhibits a substantial class imbalance, as binding residues constitute only a small fraction of the total residues. To address this challenge, we employed the Focal Loss function[10], which down-weights easy negatives and focuses learning on hard or minority positive samples.

The loss is defined as:

$$L_{\text{focal}} = -\alpha_t (1 - p_t)^{\gamma} \log(p_t)$$

where

$$p_t = \begin{cases} p & \text{if } y = 1 \\ 1 - p & \text{otherwise} \end{cases}$$

Here, p denotes the model's predicted probability for the positive class, y is the ground-truth label, α_t is a weighting factor to balance positive and negative classes, and γ controls the degree of focus on misclassified samples.

In our implementation, we set $\alpha = 0.25$ and $\gamma = 2$, following common practice in imbalanced learning scenarios. This approach effectively mitigates class imbalance and enhances model sensitivity to binding residues without sacrificing specificity.

References

1. Selvaraju, R. R. *et al.* Grad-CAM: Visual Explanations from Deep Networks via Gradient-Based Localization. in *2017 IEEE International Conference on Computer Vision (ICCV)* 618–626 (IEEE, Venice, 2017). doi:10.1109/ICCV.2017.74.
2. Liu, T., Lin, Y., Wen, X., Jorissen, R. N. & Gilson, M. K. BindingDB: a web-accessible database of experimentally determined protein-ligand binding affinities. *Nucleic Acids Res* **35**, D198-201 (2007).
3. UniProt Consortium. UniProt: a worldwide hub of protein knowledge. *Nucleic Acids Res* **47**, D506–D515 (2019).
4. Varadi, M. *et al.* AlphaFold Protein Structure Database: massively expanding the structural coverage of protein-sequence space with high-accuracy models. *Nucleic Acids Research* **50**, D439–D444 (2022).
5. Kim, S. *et al.* PubChem Substance and Compound databases. *Nucleic Acids Res* **44**, D1202-1213 (2016).
6. Morris, G. M. *et al.* AutoDock4 and AutoDockTools4: Automated docking with selective receptor flexibility. *J Comput Chem* **30**, 2785–2791 (2009).
7. Pronk, S. *et al.* GROMACS 4.5: a high-throughput and highly parallel open source molecular simulation toolkit. *Bioinformatics* **29**, 845–854 (2013).
8. Li, P. & Liu, Z.-P. GeoBind: segmentation of nucleic acid binding interface on protein surface with geometric deep learning. *Nucleic Acids Research* **51**, e60–e60 (2023).
9. Igashov, I. *et al.* Decoding Surface Fingerprints for Protein-Ligand Interactions. Preprint at https://doi.org/10.1101/2022.04.26.489341 (2022).
10. Lin, T.-Y., Goyal, P., Girshick, R., He, K. & Dollar, P. Focal Loss for Dense Object Detection. in *2017 IEEE International Conference on Computer Vision (ICCV)* 2999–3007 (IEEE, Venice, 2017). doi:10.1109/ICCV.2017.324.
